# Empowering TiO_2_–coated PVDF membranes stability with polyaniline and polydopamine for synergistic separation and photocatalytic enhancement in dye wastewater purification

**DOI:** 10.1038/s41598-024-66996-w

**Published:** 2024-07-10

**Authors:** Thi My Hanh Le, Rasika Chuchak, Sermpong Sairiam

**Affiliations:** 1 International Postgraduate Program in Hazardous Substance and Environmental Management, Chulalongkorn University, Bangkok, 10330 Thailand; 2 Center of Excellence on Hazardous Substance Management, Chulalongkorn University, Bangkok, 10330 Thailand; 3 Water Science and Technology for Sustainable Environment Research Unit, Chulalongkorn University, Bangkok, 10330 Thailand; 4 Department of Environmental Science, Faculty of Science, Chulalongkorn University, Bangkok, 10330 Thailand

**Keywords:** Photocatalytic membrane, Polydopamine, Polyaniline, PVDF membrane, TiO_2_, Environmental sciences, Environmental chemistry, Pollution remediation, Chemical engineering, Materials science

## Abstract

Photocatalytic membranes are effective in removing organic dyes, but their low UV resistance poses a challenge. To address this, self-protected photocatalytic PVDF membranes were developed using polyaniline (PANI) and polydopamine (PDA), whaich are anti-oxidation polymers, as interlayers between the membrane and TiO_2_. PVDF membranes were first modified by a self-polymerization layer of either PANI or PDA and then coated with titanium dioxide (TiO_2_). The TiO_2_ remained firmly attached to the PANI and PDA layer, regardless of sonication and prolonged usage. The PANI and PDA layers enhanced the durability of PVDF membrane under UV/TiO_2_ activation. After 72 h of irradiation, PVDF–PDA–TiO_2_ and PVDF–PANI–TiO_2_ membranes exhibited no significant change. This process improved both separation and photocatalytic activity in dye wastewater treatment. The PVDF–PDA–TiO_2_ and PVDF–PANI–TiO_2_ membranes showed enhanced membrane hydrophilicity, aiding in the rejection of organic pollutants and reducing fouling. The modified membranes exhibited a significant improvement in the flux recovery rate, attributed to the synergistic effects of high hydrophilicity and photocatalytic activity. Specially, the flux recovery rate increased from 17.7% (original PVDF) to 56.3% and 37.1% for the PVDF–PDA–TiO_2_ membrane and PVDF–PANI–TiO_2_ membrane. In dye rejection tests, the PVDF‒PDA‒TiO_2_ membrane achieved 88% efficiency, while the PVDF‒PANI‒TiO_2_ reached 95.7%. Additionally, the photodegradation of Reactive Red 239 (RR239) by these membranes further improved dye removal. Despite an 11% reduction in flux, the PVDF–PDA–TiO_2_ membrane demonstrated greater durability and longevity. The assistance of PANI and PDA in TiO_2_ coating also improved COD removal (from 33 to 58–68%) and provided self-protection for photocatalytic membranes, indicating that these photocatalytic membranes can contribute to more sustainable wastewater treatment processes.

## Introduction

The growing global population and rising living standards are driving up the demand for clean water resources. To tackle issues of water and energy scarcity, there’s a pressing need for energy-efficient and fast water purification techniques, aligning with Sustainable Development Goal (SDG) 6 for clean water and sanitation. Wastewater contains a mix of pollutants, including harmful organic substances that can impact human health^1^. Industries that employ excessive amounts of chemicals, particularly those in food processing, agriculture, textiles, and paper manufacturing, often discharge a significant amount of hazardous substances into the environment^2^. Some dyes are considered carcinogenic and non-biodegradable^3^, making them unsafe to discharge even minute quantities (below 1 ppm for certain dyes), as they pose risks to aquatic life and human health due to their toxicity and color.

Membrane separation techniques have gained attention for dye wastewater treatment because of their high separation efficiency, ease of use, and reduced pollution compared to traditional water-purification methods^4^. Membrane filtration is also at the forefront of wastewater treatment technology, effectively removing pigments, chemical oxygen demand (COD), and salinity^5^. Poly(vinylidene fluoride) (PVDF) membranes have been widely applied as membrane filters in scientific research and industrial processes owing to their beneficial characteristics, especially high thermal stability and chemical resistance. However, membrane fouling is the bottleneck problem in membrane separation, resulting from pollutants attaching to or depositing on the membrane’s surface or obstructing its pores, leading to decreased water flux and separation efficiency. The primary cause for membrane fouling in PVDF membranes is their hydrophobicity^6^. Several strategies have been investigated to reduce membrane fouling, including pretreatment of the feed solution, treatment process optimization, regular chemical cleaning, and membrane modification^7^. Hydrophilic membrane alteration may be the most significant advancement in antifouling properties.

Recently, the incorporation of photocatalysts into membrane surfaces, forming photocatalytic membranes, has received significant attention. The photocatalytic membrane can be prepared by mixed matrix modification or surface coating. This process not only increases the membrane hydrophilicity but also enhances the photochemical reactivity, which degrades the foulant during filtration^8^. When applying the photocatalytic membrane, while the permeate passes through the membrane pores, organic compounds, bacteria, and viruses are captured on the membrane surface; these foulants are degraded by the photocatalytic layer, demonstrating their self–cleaning properties. To prepare photocatalytic membranes, most previous research groups chose titanium dioxide (TiO_2_) as a photocatalyst, considering its long electron–hole pair lifetime, stability across a wide pH range, strong catalytic activity, and well–known features as a cost–effective material^1^.

Photocatalytic ultrafiltration (UF) membranes have been incorporated with TiO_2_ to enhance their antifouling properties by improving their hydrophilicity^9–11^. Alias et al. modified ceramic UF membranes by TiO_2_ dip–coating; the membrane showed effective self–cleaning properties, and the humic acid rejection efficiency reached 98.56%^12^. Chen et al. modified PVDF membranes using plasma–induced poly(acrylic acid) polymerization followed by TiO_2_ coating for oily–produced water treatment; the water contact angle (WCA) declined from 83.5° to 9.1°, and the oil separation efficiency was higher than 92%^13^. Even though there is a growing amount of literature on these membranes, their practicability is a concern because of their low UV resistance and low binding stability between membrane and nanoparticles (NPs), which might cause the leak of NPs into the environment^14^. Therefore, developing a self–protected photocatalytic membrane with high NPs binding stability is reasonable for prolonged wastewater separation. Recently, radical scavengers, such as polydopamine (PDA) and polyaniline (PANI), have been used in self–protected photocatalytic membranes^8,15,16^. With its abundance of amine and catechol groups, PDA is a promising bioinspired adhesion chemical for the modification of membrane surfaces, such as polysulfone (PSf), polyethylene (PE), polycarbonate (PC), polytetrafluoroethylene (PTFE), and polyvinylidene fluoride (PVDF) membranes, via polymerization of dopamine under alkaline conditions^17^. Feng et al. modified the PSf surface by dopamine self–polymerization, followed by amine–functionalized TiO_2_ grafting; after 9 days of UV exposure, the chemical structure of PSf–PDA–TiO_2_ presented no significant change, but the membrane without PDA treatment was destroyed^8^. Wu et al. functionalized the PSf membrane matrix with TiO_2_–PDA, and the obtained membrane showed high bovine serum albumin (BSA) separation performance, beneficial self–cleaning properties, and performance stability under UV illumination^16^. PDA at low concentration was successfully applied in our previous work to improve the self-cleaning and self-protecting properties of PVDF photocatalytic membrane^18^.

Polyaniline (PANI) is a conductive polymer with benzenoid and quinonoid structural units connected to the amine– and/or imine–type nitrogen atoms through hydrogen bonding and π–π interactions. Ma et al. claimed that PANI–coated polypropylene (PP) showed high adhesion, resulting in strong bonding with metal–organic frameworks on the PP–PANI surface^19^. In addition, PANI was previously shown to block the transmission of UV irradiation to the coating substrates^15,20^. Tang et al. (2015) found that coating cotton fabric with PANI increased the UV protection factor of the pristine cotton fabric from 6.86 to 29.43^15^. In photocatalytic membranes, PANI-coated TiO_2_ integrated into mixed matrix membranes has been studied to enhance the antifouling properties^21,22^. Nawaz et al. (2021) used PANI–TiO_2_ to modify the PVDF membrane matrix and obtained a flux recovery ratio of up to 94%^23^. However, limited studies exist on a sandwich-like coating of PVDF–PANI–TiO_2_, which provides a higher surface area for TiO_2_ active sites. In addition, the role of PANI in enhancing the binding stability of TiO_2_ in PVDF membrane and membrane UV-resistant has not been well discovered.

The following work focuses on the modification of a membrane with PANI-TiO_2_. Several aspects of the membrane performance were evaluated, including anti-fouling properties, dye rejection, TiO_2_ binding stability, and UV-resistance stability. These aspects were compared to membranes modified with PDA, which was also considered an anti-oxidant polymer, for which the modifying conditions were investigated in a previous study with high stability performance^18^. PDA or PANI was self–polymerized on PVDF hollow fiber membrane surfaces; then, TiO_2_ with different concentrations was self–assembled on the membranes to obtain the photocatalytic membranes for dye wastewater treatment. Reactive Red 239 (RR 239), a mono–azo dye, was selected for the separation/photocatalytic performance experiments.

## Methodology

### Chemical and materials

PVDF hollow fiber membranes were purchased from Altrateck, China. Anatase TiO_2_ nanoparticles (NPs) (particle size of 21 nm) were obtained from Prime Nanotechnology (Thailand). Dopamine hydrochloride and tris(hydroxymethyl)aminomethane (Tris) were supplied by Sigma–Aldrich. Analine and BSA were provided by Loba Chemie, New Zealand. Ammonium persulfate and hydrochloric acid for PANI polymerization and ethanol were provided by QRec, New Zealand. Reactive Red 239 (RR 239) was supplied by DyeStar Thai Ltd. The chemical structures of PDA, PANI, and RR 239 are shown in Fig. S1. Deionized water (DI) was used in all experimental tests.

### Photocatalytic membrane preparation

Photocatalytic membrane preparation involves two processes: PDA or PANI coated on PVDF membrane surface followed by NP self–assembly. Before modification, both ends of the membranes were sealed by silicon glue, ensuring that the modification only took part in the membrane's outer surface.

#### Preparation of PDA–coated membrane

The PDA–modified PVDF membrane, denoted as PVDF–PDA was prepared as described in our previous study^18^. The details concerning PDA–coated membrane preparation are provided in Text S1.

#### Preparation of PANI–coated membrane

The PANI–coated membrane, denoted as PVDF–PANI, was prepared by adapting the procedure in the literature, directly using aniline hydrochloride and ammonium persulfate solution (APS)^24,25^. The PVDF membranes were hung in a beaker, and then 71.2 ml of aniline hydrochloride (0.1 M) was added to the beaker, followed by the addition of 50 ml of 0.125 M APS while mixing for over 2 h at 18 °C^19^. Aniline polymerized as a thin layer on the membrane surface. The modified membrane was removed and rinsed with 0.1 M of HCl and soaked in DI water 3 times to remove unreacted chemicals. Then, the modified membrane was dried for 1h at 60 °C in the oven.

#### Preparation of photocatalytic membrane

TiO_2_ NPs with concentrations ranging from 1 to 4 g/l were dispersed in 10 mM Tris buffer solution (pH 8.5) for PVDF–PDA membranes or DI water for PVDF–PANI membranes. Coating solutions were stirred using a magnetic stirrer for 30 min before agitation using an ultrasonic bath (40 kHz) at 40 °C for 1 h. Then, the PVDF–PDA and PVDF–PANI membranes were soaked in the TiO_2_ solution for 1–4 h. Treated membranes were dried at 50 °C for 15 min to speed up polycondensation before rinsing with DI water three times to eliminate unbonded TiO_2_^26^. Membranes were dried at 60 °C for 2 h before characterization. The details of membrane characterization are provided in Text S2.

#### Photocatalytic oxidation performance

The photocatalytic performances of the original PVDF, PVDF–PDA, PVDF–PANI, and PDA/PANI–TiO_2_–treated PVDF membranes were investigated. First, 20 mg/l of RR 239 was prepared in a quartz tube. A 10 cm length of PVDF hollow fiber membrane was cut and immersed in 15 ml of RR 239 solution. Then, the reactor was exposed to UV–C for 2 h, which was placed 15 cm from the lamps (254 nm, 1.35 mW/cm^2^, Phillip) to prevent the effect of heat from UV lamps^27^. A UV–vis spectrophotometer (Labomed, Spectro SC–USA) was used to measure the concentration of RR 239 at a wavelength of 542 nm. Lastly, Eq. 1 was used to compute the dye decolorization performance:

1 $$\text{Removal efficiency}\, (\%) = \frac{ {\text{C}}_{0}-\text{C}}{{\text{C}}_{0}}\times100$$

where C_0_ and C are the dye concentration (mg/l) before and after treatment.

### Separation studies

The synergistic separation/photocatalytic performance of the membranes was investigated under UV–C irradiation (2 Phillip lamps, 256 nm, 16 W). Dye separation and membrane antifouling performance were investigated under cross–flow filtration using 5 hollow fiber membranes (effective surface area: 44.35 cm^2^) placed in a quartz module, as shown in Fig. S2. All experiments were carried out at a constant operating pressure of 1 atm and a water flow rate of 19 l/h.

#### Membrane fouling

Membrane fouling was investigated using BSA solution as a protein model. First, membranes were compacted using distilled water at the pressure of 1 atm for 30 min, and then the pure water fluxes (JW_0_) of the membranes were recorded and computed using Eq. 2.

2 $$J= \frac{V}{A\times \Delta t}$$

where J is the water flux (l/m^2^ h), and V is the permeate volume (l) passing through an effective membrane surface A (m^2^) in a filtration time Δt (h).

The feed concentration of the BSA solution was 500 mg/l, and the filtration process was continued for 30 min. Then, the BSA permeate was collected while the retentate was recycled back to the feed tank. BSA solution was color developed by modified-Lowry protein assay and measured using a spectrophotometer at 750 nm^28^. The BSA rejection rate was calculated using Eq. 3.

3 $$R=\frac{{C}_{f}-{C}_{p}}{{C}_{f}}\times 100\%$$

where C_p_ is the concentration of the permeate solution (mg/l), and C_f_ is the concentration of the feed solution (mg/l).

After rinsing the membrane modules with a water–ethanol mixture for ten minutes, J_W1_ was measured by repeating the pure water flux. The membrane antifouling performance was evaluated based on flux recovery rate (FRR), calculated using Eq. 4.

4 $$\text{FRR = }\frac{{\text{J}}_{\text{w1}}}{{\text{J}}_{\text{w0}}}\times {100\%}$$

#### Dye separation study

First, a peristaltic pump (L/S®Easy–load®II, Masterflex) was used to feed 50 mg/l of RR 239 into the quartz module and operated for 30 min in dark mode to reach equilibrium. After that, UV–C light was switched on to activate photocatalytic oxidation on the membrane surface. The permeate was collected in 3 h for evaluation of the permeate flux, dye rejection, and COD removal. Equation 2 was used to calculate the water flux, and Eq. 3 was used to calculate the removal efficiency of RR 239.

## Results and discussion

### Membrane modification

#### SEM results

**Figure 1:**
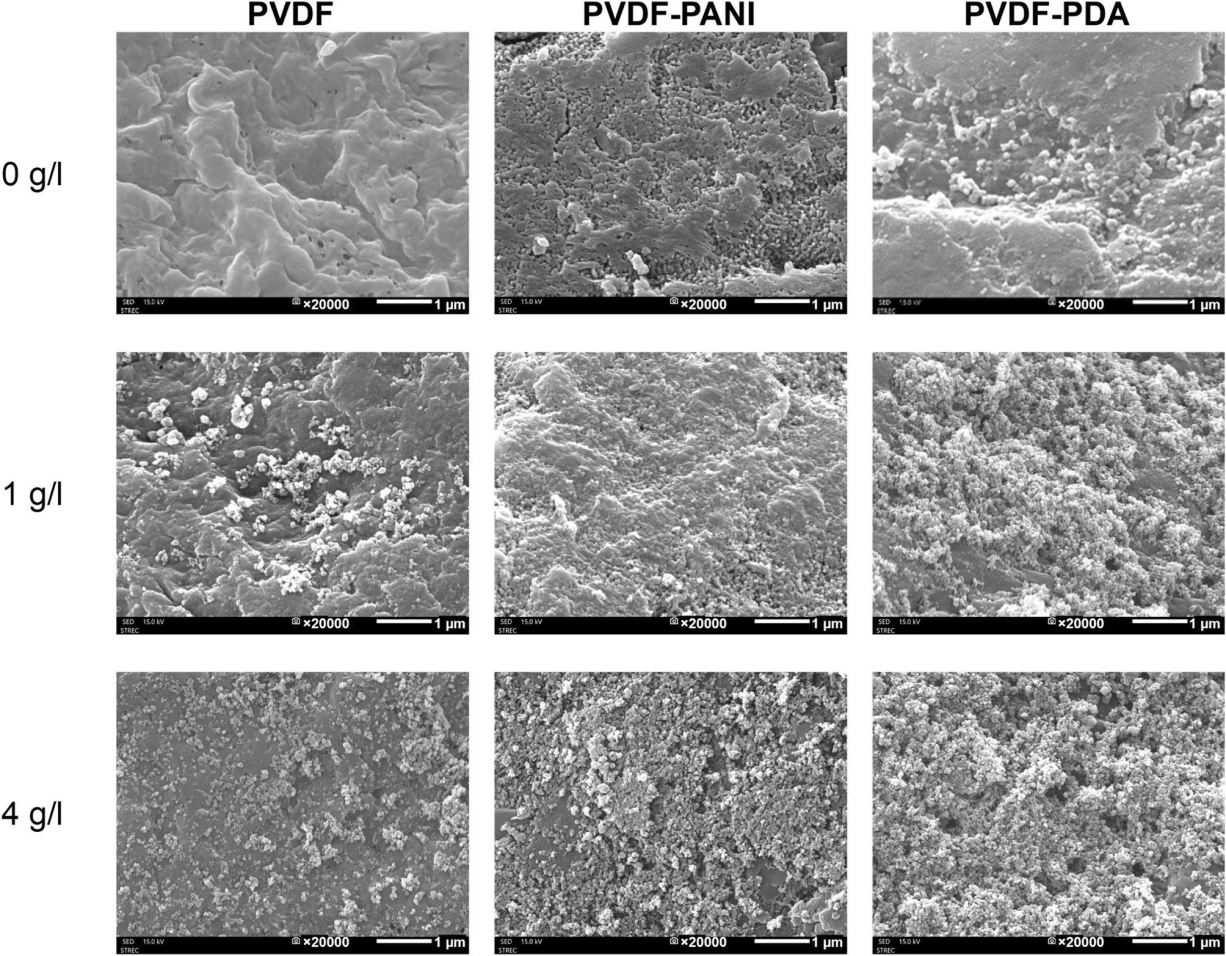
Outer surfaces of the original membranes and modified membranes at different TiO_2_ concentrations (TiO_2_ coating time of 2 h); PVDF and PVDF–PDA at 0 g/l were reproduced from our previous work^18^.

**Figure 2:**
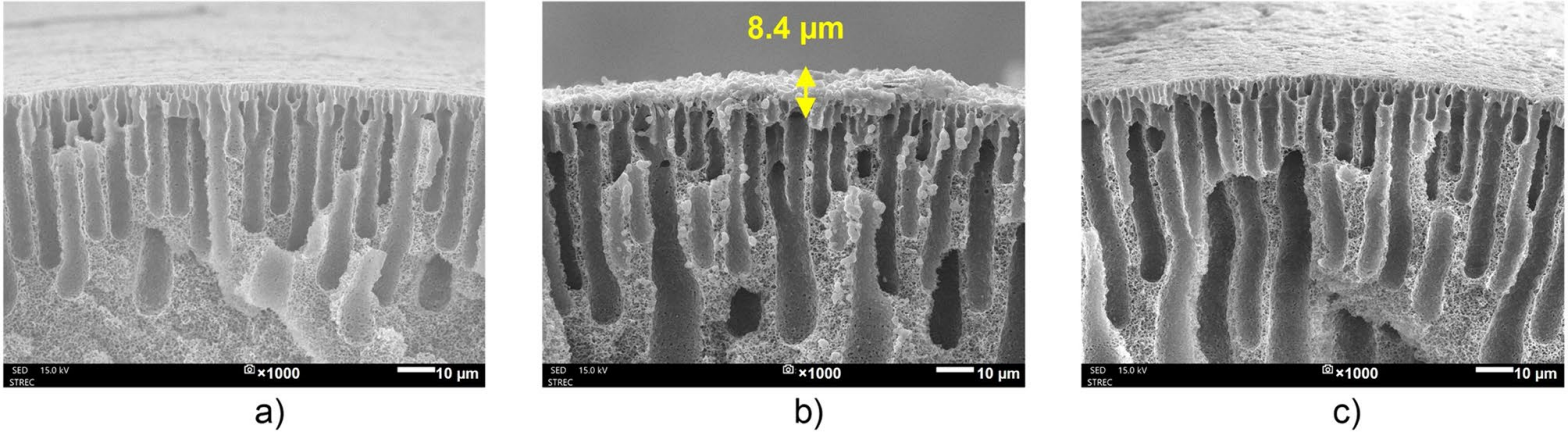
SEM images of membrane cross–sections: (**a**) original membrane, (**b**) PVDF–PANI membrane, and (**c**) PVDF–PDA membrane.

Figure 1 presents the surface morphology and Fig. 2 shows the cross-section of the original PVDF, PVDF–PDA, and PVDF–PANI membranes. Color changes indicate the successful PDA and PANI coating processes: PVDF–PANI turned dark green, and PVDF–PDA became pale brown, matching the colors of PANI emeraldine salt and PDA, as shown in previous studies^25,29^. Scanning electron microscopy (SEM) showed that the original PVDF had a smooth surface, while PVDF–PDA and PVDF–PANI exhibited rougher textures, with PVDF–PANI being rougher. After TiO_2_ coating, TiO_2_ nanoparticles (NPs) were evenly distributed on PVDF–PDA but more clustered on PVDF–PANI, with aggregate sizes of approximately 0.9–2.02 µm (Fig. S5a). Details on the NP size distributions on the membrane surfaces are described in Text S3. The cross-sections revealed that the PANI coating was about 8.4 µm thick, significantly thicker than the ultrathin PDA layer, as shown in Fig. 2. This thickness difference is due to the faster polymerization kinetics of PANI compared to PDA^30^. In addition, the reaction of PDA occurs both on substrates and in solution, whereas PANI polymerization proceeds more effectively on the substrate owing to the faster polymerization of adsorbed oligomers than from the corresponding oligomers in the solution^31^. PANI particles were also found in the pores of the PVDF–PANI membrane, as shown in Fig. 2b.

As depicted in Fig. 1, after coating with TiO_2_ (1 g/l, 2 h), TiO_2_ clusters formed on the PVDF membrane without cross-linking chemicals, but more uniform layers of TiO_2_ were formed on both the PVDF–PANI and PVDF–PDA membranes. Energy–dispersive X–ray spectroscopy was performed to obtain compositional maps, revealing the TiO_2_ distributions on the PVDF–PANI–TiO_2_ (Fig. S6) and PVDF–PDA–TiO_2_ membranes (Fig. S7). The O (cyan) and Ti (yellow) elements were distributed evenly on the PDA–TiO_2_–treated membrane, but on the PANI–TiO_2_–treated membrane, larger aggregates of O and Ti appeared with brighter colors.

To study the effect of TiO_2_ concentration on membrane modification, PVDF–PDA and PVDF–PANI membranes were immersed in TiO_2_ solutions with different concentrations, ranging from 1–4 g/l, for 2 h. As depicted in Fig. 1, more TiO_2_ was attached to the membrane surface as the TiO_2_ concentration increased.

#### Membrane hydrophilicity

**Figure 3:**
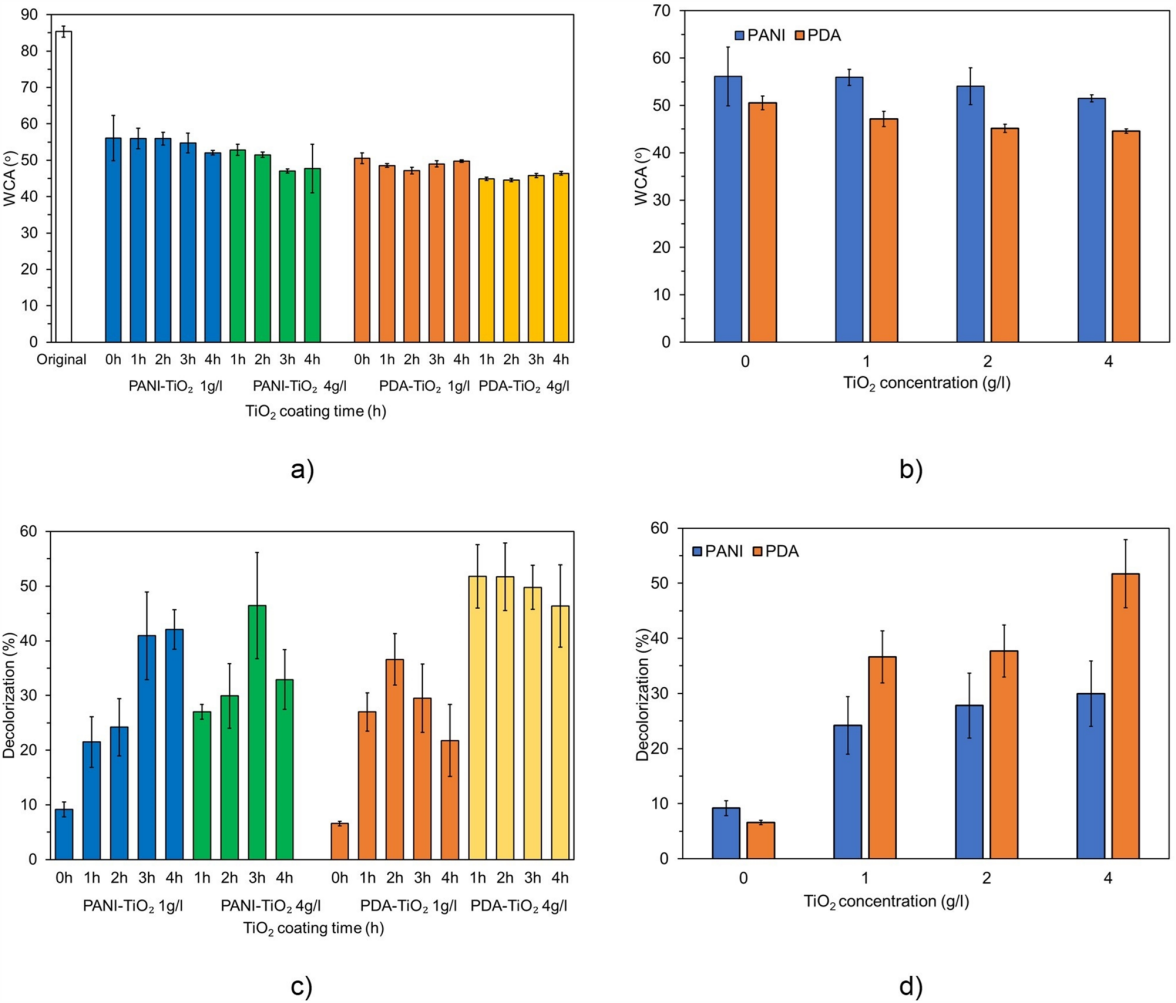
Effect of TiO_2_ coating conditions on the WCAs and membrane decolorization performances of PVDF–PDA and PVDF–PANI membranes: (**a**, **c**) TiO_2_ coating time and (**b**, **d**) TiO_2_ concentration (TiO_2_ coating time: 2 h).

As demonstrated in Fig. 3a, the WCA of the original PVDF membrane decreased following treatment with PDA and PANI, from 85.37° to 50.53° and 56.09°, respectively, indicating that PDA and PANI completely covered the PVDF membrane. The abundance of amine and hydroxyl–rich groups in PDA and PANI was linked to increased membrane hydrophilicity^21,29^. In particular, PANI introduced the –NH group on the surface of the membrane, which has the tendency for hydrogen binding, enhancing hydrophilicity^21,23^. Similarly, hydrophilic groups (–OH and –NH–) from PDA have been successfully deposited on PVDF surfaces, resulting in a lower WCA^32^. Herein, PVDF membranes pretreated with PDA show higher hydrophilicity compared with PANI–pretreated membranes. Furthermore, after being treated with TiO_2_ (4 g/l) for 1 h, the WCA was 52.8° for the PVDF–PANI membrane and 44.8° for the PVDF–PDA membrane. Thus, the cross-linking chemical plays an important role in enhancing membrane hydrophilicity.

As depicted in Fig. 3a, PVDF–PDA and PVDF–PANI membranes' WCAs were lowered after coating them with TiO_2_, a super hydrophilic modifier. Additionally, the TiO_2_ assembly time significantly affected the membrane hydrophilicity. For the PVDF–PANI membrane, the WCA decreased with the increasing TiO_2_ coating time until it became stable. At the TiO_2_ concentration of 1 g/l, the WCA of the PVDF–PANI membrane reduced from 56.1° (1–h coating) to 52.0° (4–h coating). A similar result was observed for the TiO_2_ concentration of 4 g/l, in which the WCA reached the lowest WCA of 47.0° after coating for 3 h, then remained unchanged after further treatment. Conversely, the WCA of the PVDF–PDA membrane was increased when coating time exceeded 2 h. For the TiO_2_ concentration of 4 g/l, the WCA of the PVDF–PDA membrane declined from 50.5° (PVDF–PDA) to 44.6° after coating for 2 h. The membrane hydrophilicity correlated directly to the quantity of TiO_2_ attached to the membrane surface. This was probably because both PDA and PANI have no chemical reaction with TiO_2_, but a PVDF–PDA membrane with enhanced adhesion can bond well with TiO_2_ in a short treatment time (1 h), and PVDF–PANI with high electrical conductivity and a high surface area extended the TiO_2_ attaching time^33^.

As seen in Fig. 3b, the TiO_2_ concentration has a significant influence on membrane hydrophilicity. The WCA decreased with the increase in TiO_2_ concentration. For the TiO_2_ coating time of 2 h, when the TiO_2_ solution concentration increased from 1 to 4 g/l, the WCAs of the PVDF–PDA and PVDF–PANI membranes decreased from 47.14° to 44.56° and from 55.9° to 51.5°, respectively. As previously mentioned, a higher Ti percentage was detected in membranes coated using a higher TiO_2_ concentration, resulting in more hydroxyl groups on the membrane surface and a decrease in the WCA^34^. At a TiO_2_ concentration of 4 g/l, after being treated for 3 h, the PVDF–PANI membrane exhibited the lowest WCA of 47.0°, whereas the optimized coating time for the PVDF–PDA membrane was 2 h, reaching a WCA of 44.6°.

#### Photocatalytic activities of the PVDF–PDA–TiO_2_ and PVDF–PANI–TiO_2_ membranes under UV light

The decolorization of RR 239 by photooxidation was investigated with a dye concentration of 20 mg/l at room temperature for 60 min. The RR 239 removal rates of PVDF–PDA and PVDF–PANI membranes with different TiO_2_ concentrations and coating times are shown in Fig. 3c–d. The PVDF–PANI and the PVDF–PDA membranes slightly decomposed RR 239. Only 6.57% of RR 239 was removed by the PVDF–PDA membrane within 1 h of UV irradiation, and 9.19% was removed by the PVDF–PANI membrane. The degradation efficiency mainly depended on the photolysis process, and the higher dye removal of PVDF–PANI was due to the high adsorption capacity of PANI, as previously reported^35^.

After being treated with TiO_2_, the photooxidation activity of the membranes increased rapidly owing to the catalyst properties resulting in •OH generation. •OH possesses high breaking-down power that can degrade pollutants into smaller molecules and achieve mineralization (CO_2_ and H_2_O), resulting in dye decolorization. The distinction in decolorization efficiency of PVDF–PDA and PVDF–PANI membranes after further treatment with TiO_2_ by different coating times was investigated using TiO_2_ concentrations of 1 and 4 g/l. At the lower TiO_2_ concentration (1 g/l), PVDF–PDA membranes gave higher decolorization performance than PVDF–PANI membranes for the first 2 h; however, the PVDF–PANI membranes showed better efficiency after additional coating time. The decolorization efficiency of the PVDF–PDA membranes increased from 26.98 to 36.61% when the TiO_2_ assembly time was changed from 1 to 2 h, then decreased to 27.71% when the time reached 4 h. In contrast, the RR 239 removal increased with the TiO_2_ coating time for the PVDF–PANI membrane. The efficiency of the PVDF–PANI membrane was enhanced from 21.49% (1 g/l, 1 h) to 42.1% (1 g/l, 4 h). When the TiO_2_ solution concentration increased to 4 g/l, the highest decolorization performance shifted to a shorter coating time in both the PVDF–PDA and PVDF–PANI membranes. For PVDF–PDA membranes, the decolorization efficiency was maintained at 51.8% after being coated by TiO_2_ for 1–2 h, then reduced to 46.36% after coating for 4 h. For PVDF–PANI membranes, when the TiO_2_ assembly time changed from 1 to 3 h, the dye removal efficiency increased from 27.0% to 46.5%, but it decreased to 32.9% after coating for 4 h. These results were directly correlated with the membrane hydrophilicity, illustrated in Sect. “Membrane hydrophilicity”. This implies that for membranes treated with the same cross-linking chemical, the hydrophilicity depends on the quantity of TiO_2_ assembled on the membrane surface. As a result, in this work, the membrane hydrophilicity shows a positive correlation with their photooxidation efficiency.

The photocatalytic activities with different TiO_2_ coating concentrations were investigated (Fig. 2d). The performance of the PVDF–PDA–TiO_2_-2 h membrane shows a significant change with the change in TiO_2_ concentration, reduced from 51.7 to 36.6% when the coating time was reduced from 4 to 1 g/l. This resulted from higher catalyst loading, which raised the quantity of active sites and improved light penetration on the surface of the photocatalyst. Thus, there was an increase in the number of hydroxyl radicals (•OH), which caused more RR 239 to be broken down by •OH^34^. RR 239 removal rate of PVDF–PANI–TiO_2_-2h membranes slightly declined from 29.9 to 24.2%, suggesting that TiO_2_ concentration has an insignificant impact on the performance of the PVDF–PANI–TiO_2_ membrane. This was probably due to the lower adhesive property of PANI compared to PDA, that potentially enable it to accommodate a specific amount of TiO_2_ on its surface.

In summary, TiO_2_-coated membranes, with the help of PDA and PANI, increased the membrane hydrophilicity and photocatalytic oxidation performance, which helped prevent membrane fouling. The TiO_2_ concentration of 4 g/l with 1 h of coating was optimal for the PVDF–PDA membrane (PVDF–PDA–TiO_2_), whereas 3 h was optimal for the PVDF–PANI membrane (PVDF–PANI–TiO_2_). These optimized membranes were further characterized, as described in Sect. “Characterization of optimized membranes”.

### Characterization of optimized membranes

#### Chemical structure characterization

##### FT–IR

**Figure 4:**
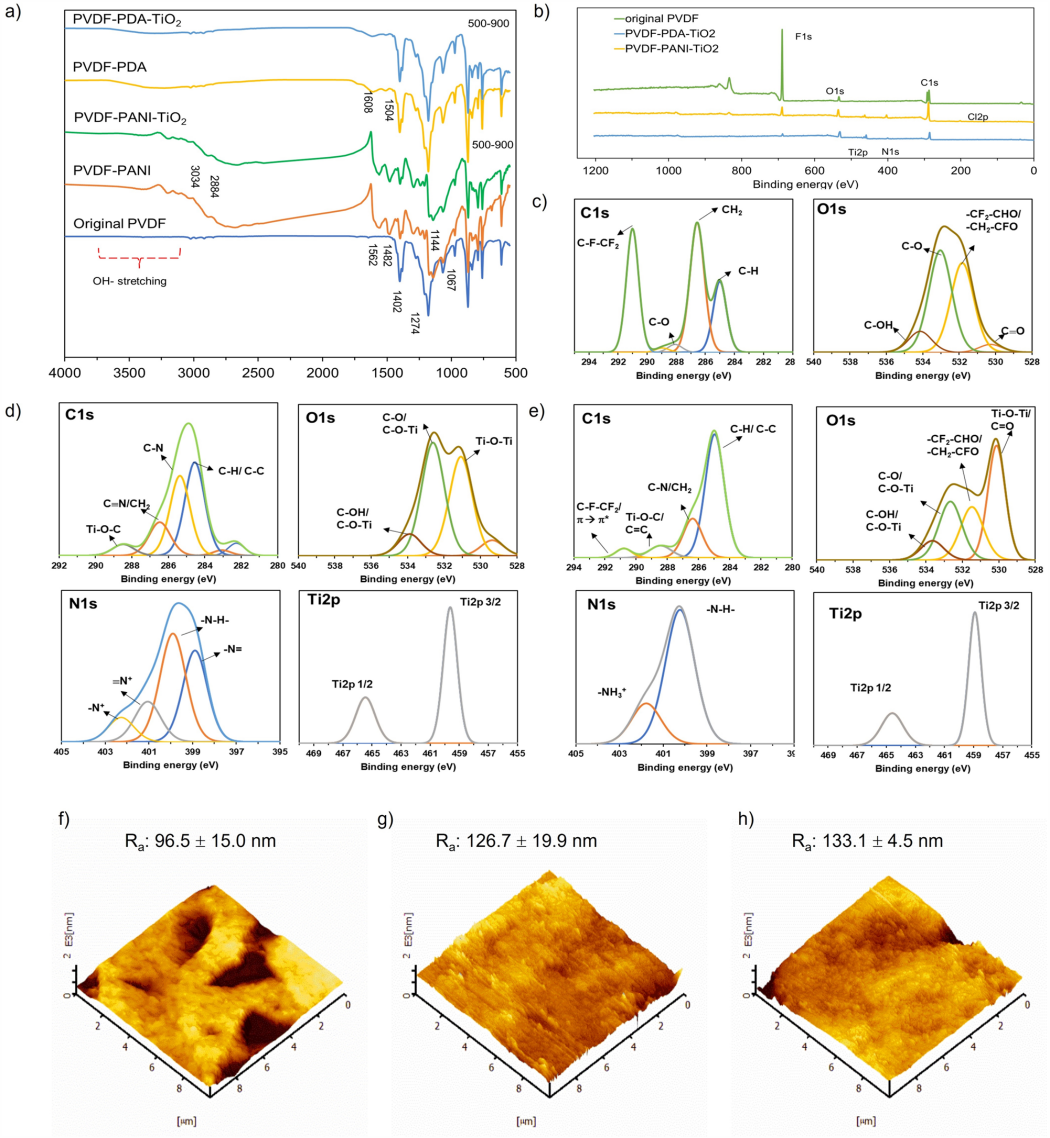
Characterization of original PVDF and optimized membranes: (**a**) FT–IR spectra of original and modified membranes; (**b**) XPS survey; and XPS spectra and AFM 3D images of (**c**, **f**) original PVDF, (**d**, **g**) PVDF–PANI–TiO_2_, and (**e**, **h**) PVDF–PDA–TiO_2_.

Fourier–transform infrared (FT–IR) spectroscopy was used to confirm the functional groups of the membranes. The features of PVDF, CF_2_ stretching (1067 cm^−1^) and CH_2_ group (1274 cm^−1^), were observed on all membranes (Fig. 4a)^36^. For both the PVDF–PDA and PVDF–PANI membranes, a broad band in the range of 2900–3500 cm^−1^ appeared, which corresponded to the addition of − OH overlapped with a stretching vibration and N–H stretching on the membrane surface, originating from functional groups in PDA and PANI^37^. For the PVDF–PANI membrane, bands at 2884 cm^−1^ and 3034 cm^–1^ were due to the symmetric and asymmetric C–H stretching vibrations, respectively^38^. Furthermore, the C=C stretching modes of quinonoid (Q) and benzenoid (B) were linked to the bands at 156_2_ and 1482 cm^−1^, respectively, while the C–N stretching vibrations of B–N+H=Q and B–N+H–B were attributed to the band at 1140 cm^−1^ in the PANI emeraldine salt^39^. A peak at 1504 cm^−1^ in the PVDF–PDA membrane indicated the combination of aromatic C=C stretching and NH bending, while a peak at 1608 cm^−1^ indicated the C=O in the amide group, which came from PDA^40^. These results confirm that PDA and PANI were coated on the PVDF membrane surfaces. Treatment with TiO_2_ resulted in a wide peak in the range of 500–900 cm^−1^, which corresponds to Ti–O vibrations in the crystal structure. Figure S8 shows a side-by-side spectra comparison of original and modified membranes. It can be seen that deeper peaks were observed on PVDF–PDA and PVDF–PANI membranes in the range of 500–900 cm^−1^ after being treated with TiO_2_ and deepened with TiO_2_ concentration. PVDF–PDA treated with 4 g/l TiO_2_ shows a lower transmittance percentage of this range compared to membrane treated with 2 g/l TiO_2_. In addition, the strong peaks in the feature spectra of PDA and PANI were reduced, implying that TiO_2_ covered the PDA and PANI layers.‬‬

#### XPS

X–ray photoelectron spectroscopy (XPS) was employed to further analyze the chemical composition of membranes. As shown in Fig. 4b, the broad scan spectrum of PVDF showed the presence of C, F, and O, whereas N and Ti appeared in membranes treated with PDA–TiO_2_ and PANI–TiO_2_. Notably, the results show that only C, F, and O were present in the original membrane (Table 1). Owing to the presence of amine groups in PDA and PANI, N appeared on PVDF–PDA and PVDF–PANI membranes. In addition, trace Cl was observed on the PVDF–PANI membrane, originating from dopant HCl in the PANI emeraldine salt^41^. The O concentration increased dramatically from 4.52 (original membrane) to 26.1% and 10.8% in PVDF–PDA–TiO_2_ and PVDF–PANI–TiO_2_ membranes, respectively, due to the addition of TiO_2_ on the membrane surfaces. Moreover, the Ti detected on PDA (14.3%) was higher than that on PANI (4.8%), probably owing to the homogenous coating of TiO_2_ on the PVDF–PDA membrane surface. Additionally, the F content decreased from 50.1% (original membrane) to 7.18% of the PVDF–PDA–TiO_2_ membrane and 11.1% of the PVDF–PANI–TiO_2_ membrane, probably owing to the complete coverage of the coating layer on the membrane surfaces.

**Table 1:** Characterizations of original, PVDF–PDA–TiO_2_ and PVDF–PANI–TiO_2_ membranes.

Membranes	WCA (°)	Membrane porosity (%)	Elemental composition (mass %)					
			C	N	O	F	Cl	Ti
Original PVDF	85.5*	68.8	44.5	–	4.52	51.0	–	–
PVDF–PDA	50.5*	78.2						
PVDF–PANI	56.1	80.4						
PVDF–TiO_2_	51.6	84.0	40.4	–	7.0	48.8	–	3.8
PVDF–PDA–TiO_2_	44.8	82.5	49.0	3.5	26.1	7.2	–	14.3
PVDF–PANI–TiO_2_	47.0	85.4	62.6	6.5	10.8	11.1	2.7	4.8

*The results were reported in our previous work^18^.

To further clarify the contribution of the functional groups, the high–resolution spectra of the original PVDF, PVDF–PDA–TiO_2_, and PVDF–PANI–TiO_2_ membranes, including the C 1s, O 1s, N 1s, and Ti 2p core level spectra, were analyzed, as illustrated in Fig. 4b–e and Table S1. Regarding the original PVDF membrane, the peaks at 291.0 eV for C 1 s were attributed to C–F–(CF_2_), and CH_2_ was identified at 285.0 eV (C 1s). Furthermore, C–H (285.0 eV), C–O (289.0 eV), C=O (530.3 eV), and C–OH (541.1 eV) were observed. As shown in Table 1, the C concentration increased from 44.5% (original membrane) to 49% and 62.6% after PDA–TiO_2_ and PANI–TiO_2_ treatment, respectively, attributed to PDA catechol groups and the PANI backbone ^17,42^. C–F–(CF_2_) disappeared for the PVDF–PANI–TiO_2_ membrane, and only a weak peak (290.8 eV) was found on the PVDF–PDA–TiO_2_ membrane, due to the coatings covering the PVDF. In addition, C–C (overlapped with C–H), C–N (285.3 eV), C≡N (overlapped with CH2), and C–O–Ti (288.5 eV) were observed for the PVDF–PANI–TiO_2_ and PVDF–PDA–TiO_2_ membranes, owing to the introduction of amine and TiO_2_ to the membrane surfaces^17,43^.

The N 1 s regions for the PVDF–PDA–TiO_2_ and PVDF–PANI–TiO_2_ are shown in Fig. 4d and e, and the attributed functional groups are listed in Table S1. The species of − N + (398.9 eV) and ≡N+ (402.3 eV) indicate the interaction between the N in PANI and the O of TiO_2_ in the PVDF–PANI–TiO_2_ membrane^44^. The strong peak at 400.2 eV in PVDF–PDA–TiO_2_ and 399.9 eV in PVDF–PANI–TiO_2_ were assigned to interstitial N species bonding with the lattice O in the host TiO_2_^45^. Additionally, the peak at 401.8 eV in the PVDF–PDA–TiO_2_ membrane was assigned to C–NH_2_ groups, suggesting the presence of quaternary ammonium compounds and validating the successful synthesis of PDA films^46^.

In the Ti2p spectra, signals corresponding to Ti2p 3/2 and Ti2p 1/2 were detected. The Ti2p 3/2 and Ti2p 1/2 peaks were observed at 458.9 and 464.6 eV for PVDF–PDA–TiO_2_ and at 459.6 and 456.4 eV for PVDF–PANI–TiO_2_^47^. As a result, there were changes in the binding energy of O 1 s. Notably, the peak at Ti–O–Ti was dominant in the modified membranes, observed at 530.1 eV on the PVDF–PDA–TiO_2_ membrane and 531.0 eV on the PVDF–PANI–TiO_2_ membranes. The C–OH functional group (534.1 eV) in the spectra of the original membrane was shifted to lower binding energy, 533.7 eV for PVDF–PDA–TiO_2_ and 533.9 eV for PVDF–PANI–TiO_2_, due to the formation of C–O–Ti after TiO_2_ coating.

#### Membrane roughness

The mean roughness (Ra) and the surface profile were studied, comprising the peak height (Z), peak diameter, and distance between peaks. Figure 4f–h shows the 3D images and average roughnesses of the original and modified membranes. The modified membranes were rougher. In addition, the roughness of PANI–TiO_2_ and PDA–TiO_2_–treated membranes were not significantly different. PANI–TiO_2_ and PVDF–TiO_2_ treatment raised the membrane’s Ra from 96.5 nm (original) to 126.7 and 133.1 nm, respectively. Moreover, the cross-sectional profile was observed by atomic force microscopy (AFM), as shown in Fig. S9. The TiO_2_ aggregates exhibited similar peak diameters and distances between peaks on the PVDF–PDA and PVDF–PANI membranes. However, from the rougher surface created by the PANI layer, as explained in Sect. "SEM results"., the peaks formed on the PVDF–PANI–TiO_2_ membrane were sharper, resulting in a higher peak height of 444 nm, compared with 138 nm of the PVDF–PDA–TiO_2_ membrane.

The increase on surface roughness of membranes after modification was related to the adhesion of TiO_2_ on membrane surface. The result was aligned with the XPS results in Sect. “Chemical structure characterization”., that higher Ti atoms presented in membrane composition was found on PVDF–PDA–TiO_2_ membrane, resulted to higher membrane roughness^48^. Additionally, the heightened sharpness of peaks, as illustrated in Fig. S9, could enhance the membrane ability to adsorb more dye molecules^48^, thereby increasing the probability of dye degradation by the •OH generated from UV/TiO_2_ activation on the membrane surface^49^. This characteristic makes the membranes promising self-cleaning membranes.

#### Mechanical strength

The mechanical strengths of the PVDF–PDA–TiO_2_ and PVDF–PANI–TiO_2_ hollow fiber membranes are summarized in Table S2. Both PVDF–PANI–TiO_2_ and PVDF–PDA–TiO_2_ membranes showed higher tensile moduli, lower strains at break, and lower tensile stresses compared with the original membrane, implying that the rigidity of modified membranes was increased while the elasticity was decreased. In addition, the elasticity of the PVDF–PDA–TiO_2_ membrane was higher than that of the PVDF–PANI–TiO_2_ membrane, but the rigidity was not significantly different. In particular, compared with the original membrane, the tensile moduli of PVDF–PDA–TiO_2_ and PVDF–PANI–TiO_2_ increased by 15.1% and 13.8%, and the strains at break were reduced by 5.3% and 7.3%, respectively. Furthermore, the tensile strengths of both the PVDF–PDA–TiO_2_ and PVDF–PANI–TiO_2_ membranes were not significantly different and were reduced by 7% compared to the original membrane. Overall, the mechanical properties of membranes did not significantly change after treatments with PDA–TiO_2_ and PANI–TiO_2_.

### TiO_2_ binding stability and UV–resistance stability

**Figure 5:**
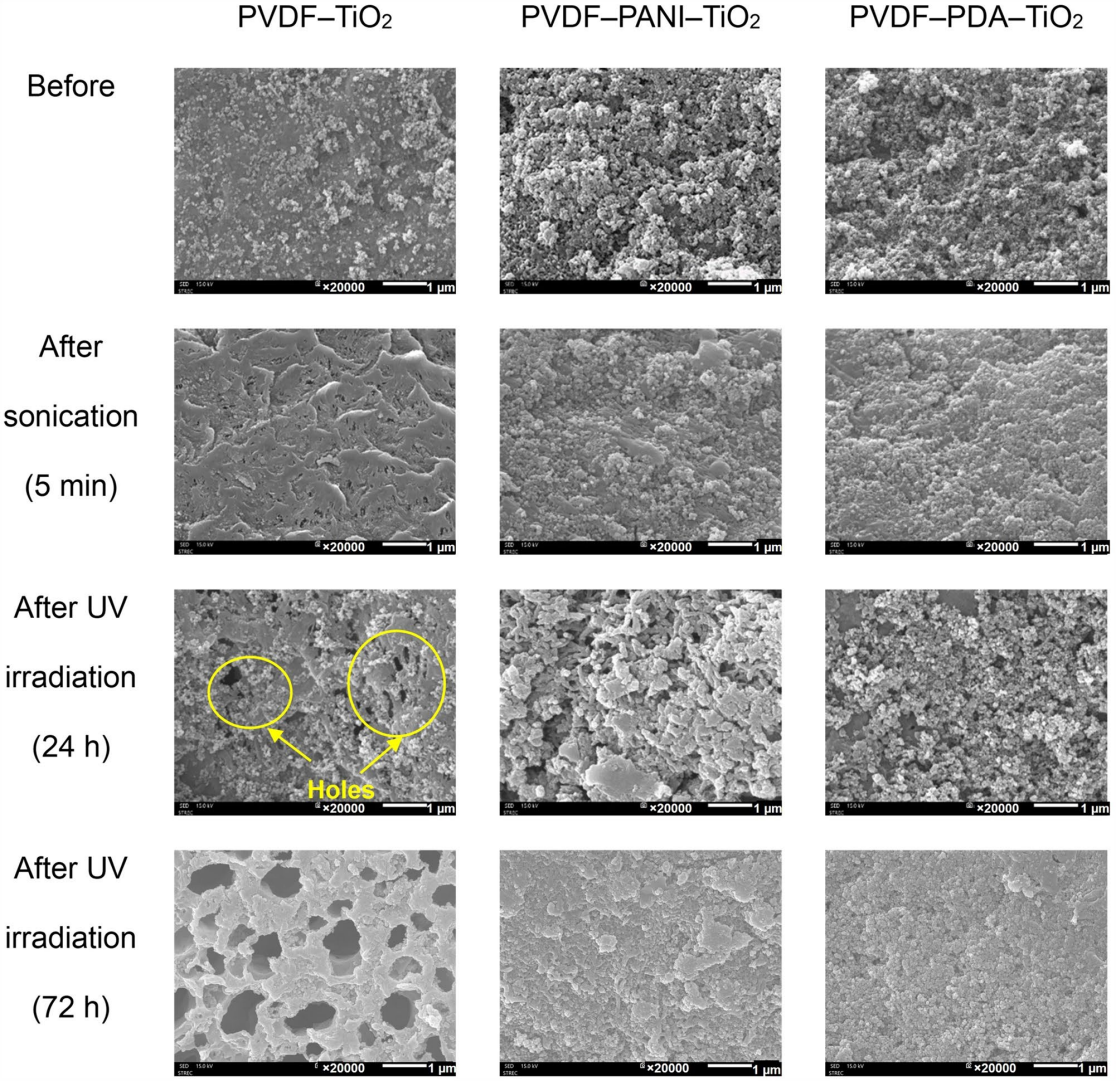
SEM images of the membrane surfaces before and after sonication (5 min) and UV irradiation (24 h).

The TiO_2_ binding stability to the membrane and their stability to UV irradiation was investigated for the PVDF–TiO_2_ membrane (without cross–linking chemical pretreatment), the PVDF–PDA–TiO_2_ membrane, and the PVDF–PANI–TiO_2_ membrane. The SEM results are shown in Fig. 5. Monolayers of TiO_2_ clusters were initially formed on the PVDF–TiO_2_ membrane, and TiO_2_ formed more uniformly on the PVDF–PDA–TiO_2_ and PVDF–PANI–TiO_2_ membranes. After interference with ultrasonic sonication for 5 min, the number and size of TiO_2_ clusters on the PVDF–TiO_2_ membrane surface were reduced, indicating that the coverage of TiO_2_ on the PVDF membrane cannot withstand ultrasonication. Compared with the PVDF–TiO_2_ membrane, the binding stabilities of TiO_2_ on PVDF–PDA–TiO_2_ and PVDF–PANI–TiO_2_ were enhanced. Although the larger clusters on the PVDF–PANI–TiO_2_ membrane were removed by sonication, more clusters remained on the surface compared with the PVDF–TiO_2_ membrane. The superior binding stability of the PVDF–PDA–TiO_2_ membrane compared to the PVDF–PANI–TiO_2_ membrane was attributed to its bioadhesive characteristics^29^.

After 24 h of UV–C exposure, the PVDF–TiO_2_ membrane surface was damaged, and some holes appeared. These holes were bigger over time, and after 72 h, the PVDF–TiO_2_ membrane was totally destroyed. Conversely, the PVDF–PDA–TiO_2_ and PVDF–PANI–TiO_2_ membranes highly resisted damage caused by UV irradiation, even after 72 h continuously irradiated by UV. PDA and PANI are radical scavengers that can protect the membrane from UV. This is because both PDA and PANI are anti-oxidant polymers^50,51^. The radical scavenger phenomena of PDA is considered similar to melanin, in which its structure serves as a theranostic antioxidant of polymers; particularly, 5,6–dihydroxyindole, a predominant component in melanin, is also found in PDA^50^. In the case of PANI, its antioxidant activity is comparable to that of phenolic antioxidants like catechin and ascorbic acid^51^; in addition, benzenoquin in PANI has been shown to have superoxide–radical scavenging activity^52^. Moreover, increasing the thickness of the membrane fiber after PANI coating can decrease the UV transmittance^15^. Herein, we found that the PVDF–PDA and PVDF–PANI layers protected the PVDF membrane from the photocatalytic activity of UV–TiO_2_.

### Performance of PVDF–PDA–TiO_2_ and PVDF–PANI–TiO_2_ membranes in water purification

The original PVDF membrane, PVDF–PDA, PVDF–PANI, PVDF–PDA–TiO_2_, and PVDF–PANI–TiO_2_ membranes were employed for separation studies, including pure water flux, membrane fouling, and dye separation studies.

#### Membrane permeability

**Figure 6:**
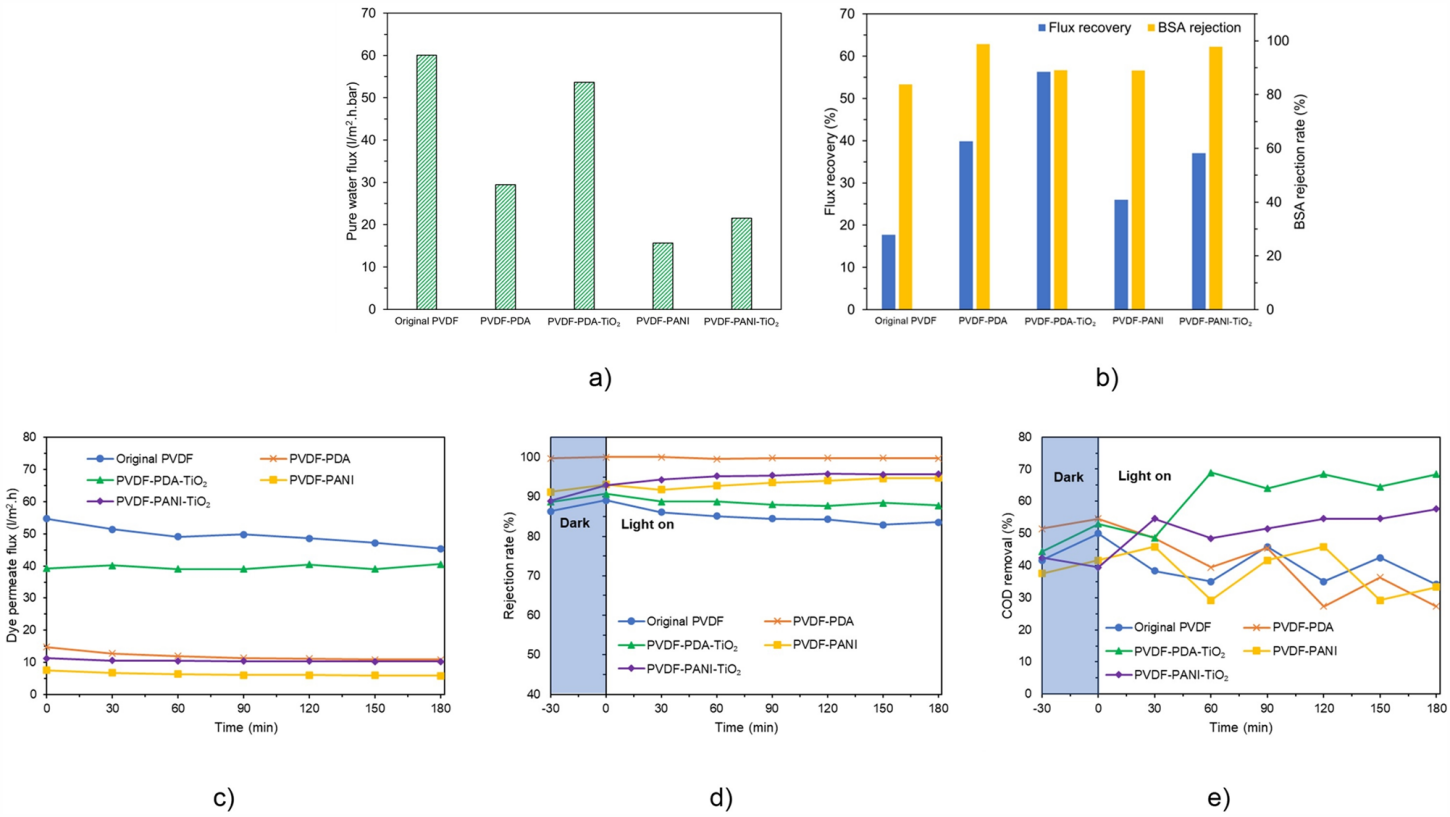
(**a**) Pure water flux and (**b**) fouling resistance and dye separation performance under UV irradiation: (**c**) dye permeate flux, (**d**) dye rejection rate, and (**e**) COD removal rate of the original and modified membranes.

The results of the pure water flux assessment are shown in Fig. 6. After treatment with PDA and PANI, the flux was significantly reduced from 60.1 l/m^2^.h of the original membrane to 29.5 and 16.7 l/m^2^.h, respectively. Davari et al. (2021) indicated that various parameters, including hydraulic resistance, porosity, and membrane hydrophilicity, determine membrane pure water flux^29^. As shown in Table 1, PVDF–PDA and PVDF–PANI membranes showed higher membrane porosity than the original membrane; the porosity of the original membrane was 68.8%, and after coating with PDA and PANI, it was increased to 78.2% and 80.4%, respectively. Nevertheless, the PDA and PANI coating layers increased the membrane hydraulic resistance, reducing the flux passing through membrane layers. In addition, the thicker layer formed by the PVDF–PANI membrane increased the hydraulic resistance, making it a lower permeate than the PVDF–PDA membrane. Similarly, Daravi et al. (2021) reported that the flux of the PES membrane was reduced by 30% after PDA treatment for 2 h^29^.

After TiO_2_ treatment, the permeate fluxes of both PVDF–PANI and PVDF–PDA membranes were higher, attributed to increased hydrophilicity and membrane porosity. As reported in Table 1, the WCAs of PVDF–PANI and PVDF–PDA membranes were reduced from 50.5° to 47.0° and from 56.1° to 44.8°, respectively, after coating with TiO_2_. In addition, the porosity of PVDF–PDA and PVDF–PANI membranes slightly increased compared with the original membrane, to 85.4% and 82.5%, respectively. As a result, the pure water flux increased to 53.7 l/m^2^.h in the PVDF–PDA–TiO_2_ membrane, which was only 10.7% lower than the original membrane, and to 21.5 l/m^2^.h in the case of PVDF–PANI–TiO_2_ membrane. Da Silva et al. (2022) reported a similar result, wherein the PVDF membrane was treated with PDA and TiO_2_ co–deposition; membranes coated with PDA–TiO_2_ exhibited an increased flux from 191 to 781 l/m^2^.h^53^. Zhang et al. (2013) coated PDA and TiO_2_ on PES membranes; the permeate flux was lost by 31% after PDA coating, then recovered by 14% after TiO_2_ treatment, resulting from increased membrane hydrophilicity^7^.

#### Membrane self–cleaning performance

The antifouling performances of the membranes regarding FRR and BSA rejection were investigated (Fig. 6b). All modified membranes revealed higher FRR and BSA rejection efficiencies than the original membrane, indicating that the antifouling of the membrane was enhanced after modification. Particularly, the FRR of the original membrane was 17.7%; that figure increased to 39.9% and 26.0% on PVDF–PDA and PVDF–PANI membranes, respectively. Daravikia et al. (2022) claimed that improving membrane hydrophilicity can increase antifouling performance^11^. Therefore, the higher FRR in the PVDF–PDA membrane compared with the PVDF–PANI membrane was due to its hydrophilicity. After TiO_2_ treatment, the FRR of the modified membrane increased with increasing membrane hydrophilicity. PVDF–PDA–TiO_2_ exhibited an FRR of 56.3%, and PVDF–PANI–TiO_2_ exhibited 37.1%. Additionally, the primary mechanism for the antifouling activity was the activation of TiO_2_ under UV irradiation; the photocatalytic activity was responsible for the foulant degradation on the membrane surface, as mentioned in Sect. “Photocatalytic activities of the PVDF–PDA–TiO_2_ and PVDF–PANI–TiO_2_ membranes under UV light”. These results agreed well with the previous studies regarding the degradation of BSA under UV/TiO_2_ activation^8,11^.

BSA rejection, as an indicator of antifouling activity, also increased after modification. According to Daravi et al. (2021), the higher membrane hydrophilicity prevented fouling attachment by forming a thin layer of water molecules on the membrane surface, which led to a higher BSA rejection^29^. Herein, pore-blocking caused by PDA, PANI, and TiO_2_ was also responsible for increasing BSA separation efficiency. The BSA rejection rate of the original membrane was 83.7%; after PDA and PANI treatment, the figure increased to 98.8% and 89.0%, respectively. After treatment with TiO_2_, the BSA rejection rate of PVDF–PDA–TiO_2_ and PVDF–PANI–TiO_2_ membranes was 89.1% and 97.8%, respectively.

#### Removal of RR239 by photocatalytic membranes

The original and modified membranes were applied in RR 239 (50 mg/l) removal. The filtration was done in the dark for 30 min before turning the light on. The original PVDF membrane's normalized flux decreased by 17.0% after 3 h of UV irradiation and the figures for PVDF–PDA and PVDF–PANI were 26.5% and 22.7%, respectively, due to pore-blocking and low adsorption^35,54^. After TiO_2_ treatment, after 3 h of irradiation, the initial flux of PVDF–PDA–TiO_2_ increased by 3.3%, while PVDF–PANI–TiO_2_'s flux loss reduced to 9.0%, indicating the antifouling properties of these membranes under UV light, as described in Sects. “Membrane self–cleaning performance” and “Photocatalytic activities of the PVDF–PDA–TiO_2_ and PVDF–PANI–TiO_2_ membranes under UV light”.

For dye separation, all membranes showed improved rejection rates in the dark due to pore-blocking caused by dye (Fig. 6d). Additionally, the rejection rate was indirectly correlated with the permeate flux. The original PVDF had the highest flux but the lowest dye rejection, which was 86.1% after 30 min and dropped to 83.5% after 3 h due to membrane fouling. PVDF–PDA had almost complete dye rejection (near 100%) over 3 h. The dye rejection performance of the PVDF–PDA–TiO_2_ membrane fluctuated during the first 30 min and then remained around 88.5%. PVDF–PANI membranes saw a steady increase in dye rejection; PVDF–PANI and PVDF–PANI–TiO_2_ achieved 94.7% and 95.7% after 3 h, respectively.

Even though the dye rejection rates of the PVDF–PDA and PVDF–PANI membranes were higher than that of the original membrane, their COD removal rates were similar and fluctuated around 33%, suggesting that the dye was merely separated, not degraded (Fig. 6e). In contrast, TiO_2_-treated membranes had higher COD removal, with rates improving over time. For example, the COD removal rate of PVDF–PDA–TiO_2_ increased from 53.1 to 68.4%, and that of PVDF–PANI–TiO_2_ rose from 39.4 to 57.6% after 3 h, confirming the effectiveness of dye decolorization by UV/TiO_2_ activation. The superior performance of PVDF–PDA–TiO_2_ was attributed to its higher photocatalytic activity, as reported in Sect. “Photocatalytic activities of the PVDF–PDA–TiO_2_ and PVDF–PANI–TiO_2_ membranes under UV light”. These results highlight the synergistic effects of membrane separation and photocatalytic degradation in dye removal applications. Specifically, when RR 239 dye, an anionic dye, passes through membranes, the membrane interlayer rejects some dye molecules, while other dye molecules can be adsorbed onto the membrane surface, which contains many active functional groups, through electrostatic interaction and creates foulants^49^. Under photocatalytic activity, these foulants are directly degraded by reactive radicals generated by UV/TiO_2_ activity , making the membrane self-cleaning and enhancing the COD removal efficiency^55^.

In summary, membrane modification by PANI–TiO_2_ and PDA–TiO_2_ not only increased the membrane separation performance but also showed effective dye decomposition. The membrane performance was more stable with the self–cleaning activity of the obtained photocatalytic membranes. Compared with the original membrane, PVDF–PDA–TiO_2_ showed a slight loss in flux (2.4%) and better dye removal performance, with increases of 4.2% in the dye rejection rate and 34.2% in COD removal; meanwhile, the flux loss in PVDF–PANI–TiO_2_ was significant (64.2%), but the dye rejection rate and COD removal were increased by 12.2% and 23.4%, respectively. In this work, PVDF–PDA–TiO_2_ membrane is more desirable for dye separation than PVDF–PANI–TiO_2_ membrane.

Membranes were taken after usage to analyze the change in their morphology and composition, as shown in Fig. S10 and Table S3. Similar to the binding stability test, after usages (6 h), PVDF–TiO_2_ was damaged with holes appearing, while PVDF–PDA–TiO_2_ and PVDF–PANI–TiO_2_ membranes remained unchanged. In addition, the change in membrane composition shows that 65.7% of Ti was detached from PVDF–TiO _2_ after using it for 6 h, while that number for PVDF-PDA-TiO_2_ and PVDF-PANI-TiO_2_ membranes was insignificant, at 4.8 and 10.4%, respectively. The results indicate that PVDF-PDA-TiO_2_ and PVDF-PANI-TiO_2_ enhanced the stability of the PVDF photocatalytic membrane.

### Comparison with other reported results in wastewater separation

The PDA– and PANI–modified membranes were compared with other modified membranes in the literature (Table 2). The findings in this work are consistent with the results reported in the previous studies. PDA– and PANI–treated membranes exhibit lower permeate fluxes compared with the original membrane, resulting in higher rejection rates. Khoo et al. (2021) modified the polyamide (PA) layer of a thin film composite reverse osmosis membrane by PANI plasma; the permeate flux was reduced by 66.3%, but the NaCl rejection rate was increased by 2%^56^. Davarikia et al. (2022) modified PVDF using polyvinyl alcohol (PVA)–TiO_2_–PDA and applied the membranes in oil/water separation; the FRR was increased from 48% (original membrane) to 87%^11^. In the present work, the PVDF–PDA–TiO_2_ membrane, with a low flux loss (2.4%) and high fouling resistance, provides effective wastewater separation.

**Table 2:** Separation performance compared with other works.

Membranes	Application	Flux loss*	FRR (%)	Rejection rate	References
PES–PVA–TiO_2–_PDA	Oil/water separation	56%	87	Increased from 62 to 92%	^11^
PES–PDA–TiO_2_	BSA separation	50%	32%	Increased from 48.1 to 84%	^29^
PA–PANI	NaCl rejection	66.3%	–	Increased from 91.4 to 93.2%	^56^
PAN–PANI	Oil rejection	33.1%	–	Increased from 90.0 to 99.8%	^57^
PVDF–PANI–TiO_2_	Dye separation	64.2%	37.1%	Increased from 83.5 to 95.7%	This work
PVDF–PDA–TiO_2_	Dye separation	10.7%	56.3%	Increased from 83.5 to 87.8%	This work

*Compared with the original membrane.

## Conclusions

PDA–TiO_2_ and PANI–TiO_2_ treatments increased the hydrophilicity of the PVDF hollow fiber membrane and demonstrated effective photocatalytic performance. The photocatalytic activity increased with the increasing TiO_2_ coating solution concentration. The PDA–TiO_2_–modified membranes showed higher hydrophilicity and photocatalytic performance compared with PANI–TiO_2_–treated membranes. PVDF–PDA–TiO_2_ and PVDF–PANI–TiO_2_ showed no significant difference in mechanical strength compared with the original PVDF membrane. PDA–TiO_2_ and PANI–TiO_2_ were strongly adhered to the PVDF membrane, and the PDA and PANI layers protected the PVDF membrane from UV/TiO_2_ activation. The modified membranes improved the antifouling properties compared with the original membrane because of the synergistic enhancement in separation and photocatalytic activity. Although the PVDF–PANI–TiO_2_ membrane exhibited a higher dye rejection efficiency, the PVDF–PDA–TiO_2_ membrane showed higher COD removal and photodegradation efficiency. Furthermore, the PVDF–PDA–TiO_2_ membrane with lower permeate flux loss (10.7% compared with 64.2% for the PVDF–PANI–TiO_2_ membrane) was identified as the best–performing membrane owing to its highly reversible fouling behavior, effective dye and COD rejection, and sufficient permeability. This study demonstrates the potential of PDA and PANI in self–cleaning photocatalytic membranes, addressing TiO_2_ leakage and UV resistance. Notably, further investigation is required to control the thickness of the PANI layer and reduce the flux loss.

### Supplementary Information

Supplementary Information.

## Data Availability

The datasets generated during and/or analyzed during the current study are available from the corresponding author upon reasonable request.
