# Supplementary material for: Empowering TiO2–coated PVDF membranes stability with polyaniline and polydopamine for synergistic separation and photocatalytic enhancement in dye wastewater purification
Source: Sci Rep. 2024 Jul 10;14:15969. doi: 10.1038/s41598-024-66996-w (PMC11237106; doi:10.1038/s41598-024-66996-w)
Supplement: Supplementary file 1 — Supplementary Information. [file 41598_2024_66996_MOESM1_ESM.docx]

**Empowering TiO_2_–coated PVDF membranes stability with polyaniline and polydopamine for synergistic separation and photocatalytic enhancement in dye wastewater purification**

*Thi My Hanh Le ^1, 2, 3^, Rasika Chuchak ^4^, and Sermpong Sairiam ^2, 3, 4*^*

^1^ International Postgraduate Program in Hazardous Substance and Environmental Management, Chulalongkorn University, Bangkok 10330, Thailand

^2^ Center of Excellence on Hazardous Substance Management,

Chulalongkorn University, Bangkok 10330, Thailand

^3^ Water Science and Technology for Sustainable Environment Research Unit, Chulalongkorn University, Bangkok 10330, Thailand

^4^ Department of Environmental Science, Faculty of Science, Chulalongkorn University, Bangkok 10330, Thailand

*Corresponding author: Sermpong Sairiam (E–mail: [*sermpong.s@chula.ac.th*](mailto:sermpong.s@chula.ac.th))

**Text S1. Preparation of PDA-coated membrane**

Briefly, the as-prepared poly(vinylidene fluoride) (PVDF) membranes were pre-wetted using ethanol, immediately immersed into the polydopamine (PDA) solution (2 g/l) prepared in Tris buffer (10 mM), and shaken at 100 rpm using an orbital shaker (Gallenkamp, England) at room temperature for 2 h. Then, membranes were removed, soaked in DI 3 times to remove unbound PDA, and dried for 5 min at 60° in an oven (Model 700, Memmert) before NP coating.

**Text S2. Membrane characterization**

Membrane hydrophilicity was analyzed by measuring the water contact angle (WCA) using a tensiometer (DCAT 11, Dataphysics, Germany) with SCAT software (version 2.4.8.48). The advancing contact angle was determined over three cycles, and to prevent the experimental measurement error, at least three sample readings of each membrane were taken. The average WCA was computed.

Scanning electron microscopy (SEM) (JSM-IT500HR, JEOL, Japan) coupled with energy-dispersive X-ray spectroscopy (EDS) was performed at an accelerating voltage of 15.0 keV to examine the morphology and chemical composition of both the unmodified and modified membranes. The membrane surface was vacuum-coated with gold prior to SEM analysis to avoid electrostatic charging. ImageJ software (version 1.53i, USA) was used to analyze membrane thickness and the particle size distributions of the adhered particles on the membranes.

To investigate any changes in the chemical functional groups, Fourier-transform infrared (FT-IR) spectroscopy was performed. Surface infrared spectra were obtained using an IR Prestige-21 FT-IR spectrophotometer (Shimadzu, Japan) in the attenuated total reflection mode, with a resolution of 4 cm^-1^. The membranes' inverted surface spectra were collected between 500 and 4000 cm^-1^.

The gravimetric method was used to determine membrane porosity (ε) [1]. Pieces of the membrane (3 cm long) were dried for 2 h at 60°C. After that, the dried membranes were weighed and then immersed in pure water for 24 h. The excess water on the membranes was wiped off, and the weight of the wetted membrane was obtained. The porosity of the hollow fiber membranes was calculated using Equation (1):

$\text{ε =}\frac{\text{(W}_{2}\text{-}\text{W}_{\text{1}}\text{)/}\text{ρ}_{\text{w}}}{\text{(W}_{\text{2}}\text{-}\text{W}_{\text{1}}\text{)/}\text{ρ}_{\text{w}}\text{+}\text{W}_{\text{1}}\text{/}\text{ρ}_{\text{p}}}$ (1)

where W_2_ and W_1_ are the weights of the weighted and dried membranes, respectively. ρ_w_ is the density of water (1 g/cm^3^), and ρ_p_ is the theoretical density of PVDF (1.78 g/cm^3^).

The mechanical strength of PVDF hollow fibers was measured before and after modification using a universal testing machine (ST Series, Tinius Olsen, GB), considering the tensile modulus and tensile stress at failure under a constant elongation at room temperature, with a strain rate of 50 mm min^−1^.

**Text S3. Membrane stability**

The membrane stability in terms of NP binding stability and UV resistance stability were investigated following the method in our previous work [2]. The study used ultrasonic treatment to test the NPs' binding capability on membrane surfaces. PVDF-TiO_2_, PVDF-PANI-TiO_2_, and PVDF-PDA-TiO_2_ membranes (5 cm in length) were placed in a 20 ml glass tube and sonicated at 25 kHz for 5 min to examine binding stability. The membranes were then removed and allowed to dry for two hours before being characterized. SEM was used to observe the changes on the membrane surface before and after ultrasonic treatment.

The stability of membranes was examined by placing 5 cm of membranes in a photocatalytic activity reactor. Subsequenlty, the membranes were placed in a dye wastewater reactor subjected to UV light for a durarion of 72 h. After testing, the membranes were removed and dried to determine surface changes.

**Text S4. Distribution of nanoparticles on membrane surface**

The distribution of particle diameters on the membrane surface was measured using ImageJ, as shown in **Figure S4**. A nano-sized microlayer of PANI was coated on the PVDF membrane surface, and a continuous film with aggregations appeared on the PVDF-PDA membrane. The mean particle size of the PVDF-PANI membrane was 91 nm, wherein 32.6% of the particles had a diameter of less than 50 nm. This result is consistent with the PANI nanoparticle size reported by Babar et al., where a nanolayer of PANI with an average size of 139.62 nm was coated on a polyurethane membrane [3]. In contrast, a smoother surface with a few large aggregations formed on the PVDF-PDA membrane (**Figure 1c**) because of the complex oligomerization process and uncontrollable self-assembled reaction that occurs through non-covalent bonds [4], resulting in 19.75% of particles having a diameter greater than 200 nm. After TiO_2_ treatment, TiO_2_ nanoparticles were distributed evenly on the PVDF-PDA membrane surface; in addition, the mean particle size was reduced to 64 nm owing to the coverage of TiO_2_-NPs on the PDA layer (**Figure S4c**). On the PVDF-PANI membranes, TiO_2_ nanoparticles aggregated with sizes of approximately 0.9–2.02 µm (**Figure S4a**).

**Table S1.** Binding energy of functional groups for each element and percentage associated with each or sum of bond type

| Elements | Functional group | Binding energy (eV) | | |
| --- | --- | --- | --- | --- |
|  |  | Original PVDF | PVDF-PDA-TiO_2_ | PVDF-PANI-TiO_2_ |
| C *1s* | C-F-(CF_2_) | 291 | 290.8 | - |
|  | C-O /  C-O-Ti | 289.0 | 288.5 | 288.5 |
|  | CH_2_/  C-N-/CH_2_ (PDA)  C≡N/ CH_2_ (PANI) | 286.6 | 286.4 | 286.5 |
|  | C-H/ C-C | 285.0 | 285.0 | 284.6 |
|  | C-N | - | - | 285.3 |
| N *1s* | N-H- | - | 400.2 | 399.9 |
|  | C–NH_2_ | - | 401.8 | - |
|  | -N= | - | - | 398.9 |
|  | ≡N^+^ | - | - | 402.3 |
| O *1s* | Ti-O-Ti/ C=O | 530.3 | 530.1 | 531.0 |
|  | C-O/ C-O-Ti | 533.0 | 532.6 | 532.6 |
|  | CH_2_-CFO/  CH_2_-CHO | 531.8 | 531.5 | - |
|  | C-OH/ C-O-Ti | 534.1 | 533.7 | 533.9 |
| Ti *2p* | Ti 2p 3/2 | - | 458.9 | 459.6 |
|  | Ti 2p 1/2 | - | 464.6 | 465.4 |

**Table S2.** Mechanical properties of original and modified PVDF membranes

| **Membrane** |  | **Tensile modulus (MPa)** | **Tensile strength (MPa)** | **Strain at break (%)** |
| --- | --- | --- | --- | --- |
| Original PVDF |  | 22.5 ± 3.2 | 1.89 ± 0.02 | 162.40 ± 5.37 |
| PVDF-PDA-TiO_2_ |  | 25.9 ± 3.7 | 1.76 ± 0.04 | 153.81 ± 6.50 |
| PVDF-PANI-TiO_2_ |  | 25.6 ± 4.5 | 1.75 ± 0.02 | 150.53 ± 9.31 |

**Table S3.** Change in composition of membranes before and after rinsing for 6 h

| Membranes | Elemental composition (mass %) | | | | | |
| --- | --- | --- | --- | --- | --- | --- |
|  | C | N | O | F | Cl | Ti |
| PVDF-TiO_2_ | 40.4 | – | 7.0 | 48.8 | – | 3.8 |
| PVDF-PDA-TiO_2_ | 49.0 | 3.5 | 26.1 | 7.2 | – | 14.3 |
| PVDF-PANI-TiO_2_ | 62.6 | 6.5 | 10.8 | 11.1 | 2.7 | 4.8 |
| After usages (6 h) | | | | | | |
| PVDF-TiO­_2_ | 56.8 | ‒ | 2.4 | 40.3 | – | 0.5 |
| PVDF-PDA-TiO_2_ | 42.3 | 4.2 | 26.6 | 13.3 | ‒ | 13.6 |
| PVDF-PANI-TiO_2_ | 64.2 | 8.8 | 11.7 | 8.5 | 2.5 | 4.3 |


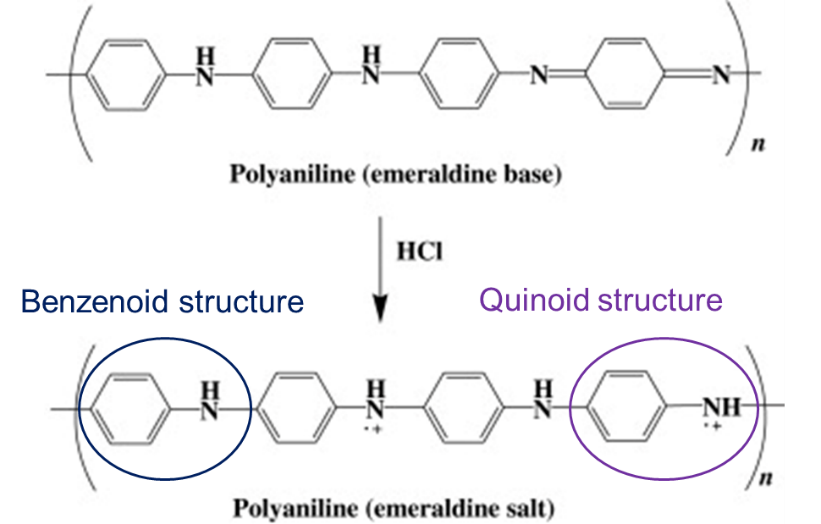


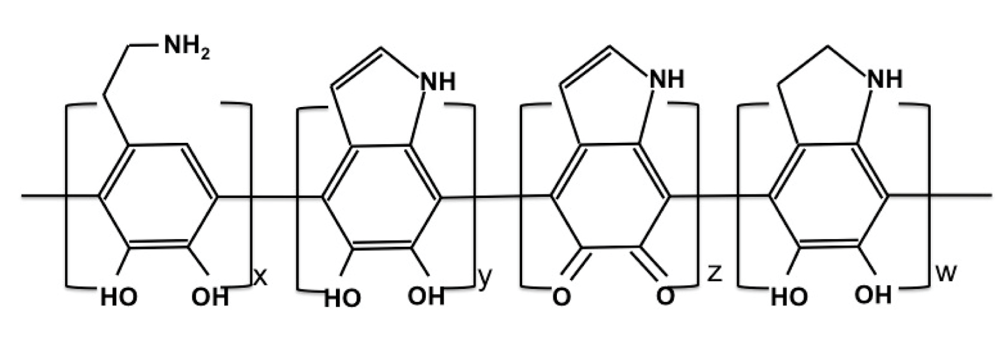


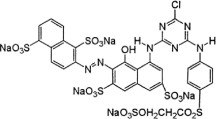


**Figure S1.** Chemical structures of a) polyaniline, b) polydopamine, and c) RR 239


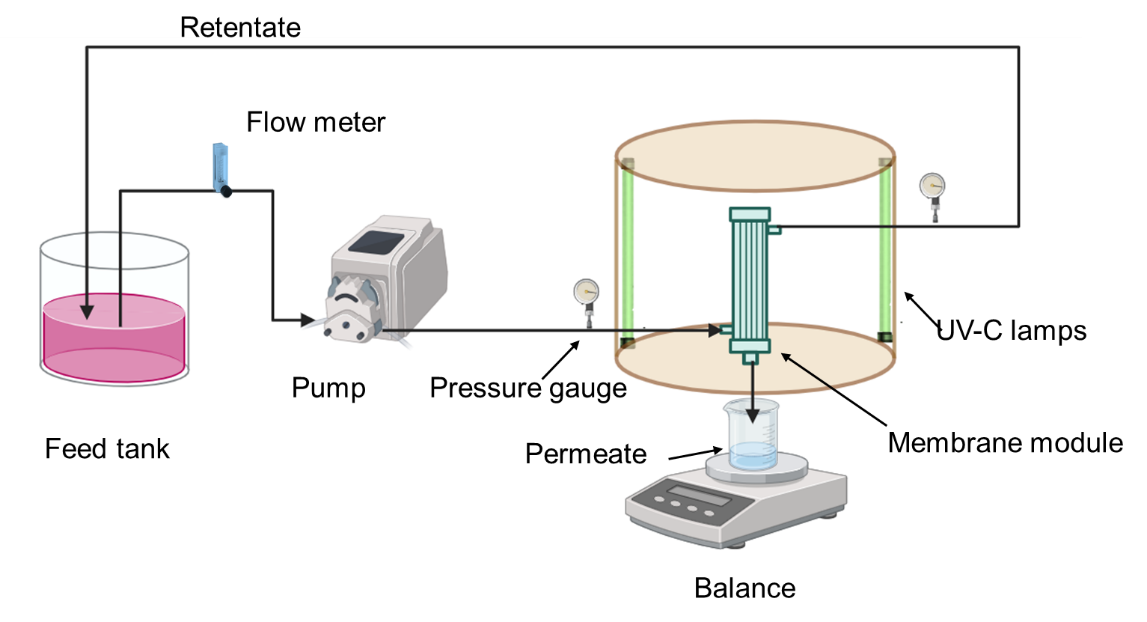


**Figure S2** Separation set up for measuring hollow fiber photocatalytic membranes in the cross-flow mode under UV irradiation

| **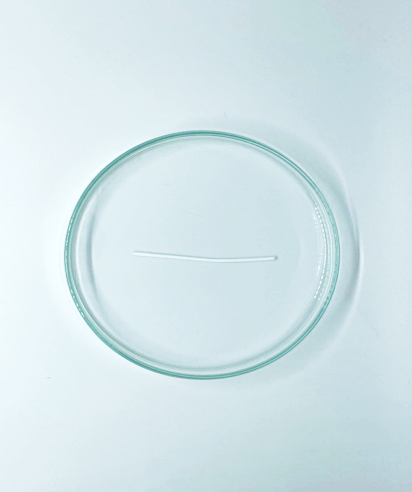** | 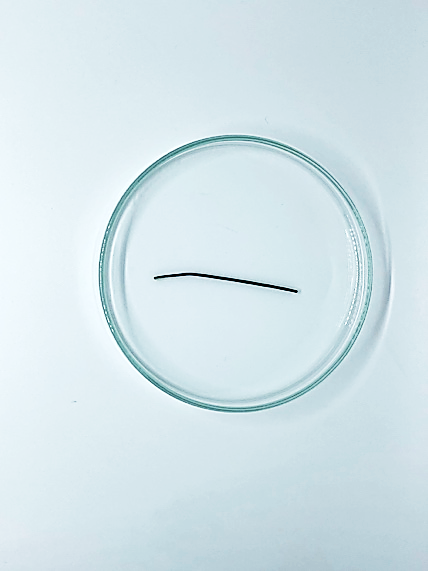 | 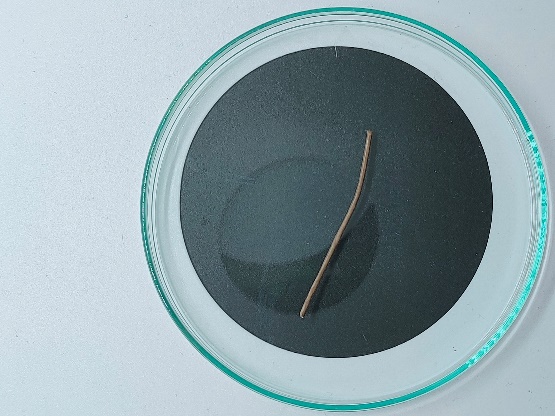 |
| --- | --- | --- |
|  |  |  |

Note: Figure S3c is reproduced from [2].

**Figure S3.** Change in the color of membranes: a) original PVDF, b) PVDF-PANI, and c) PVDF-PDA

| 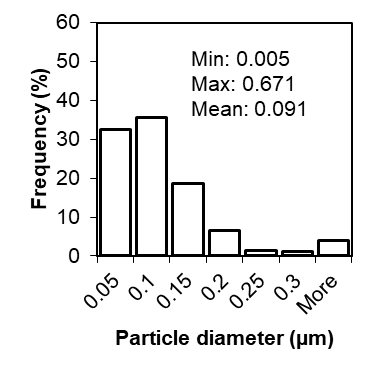 | 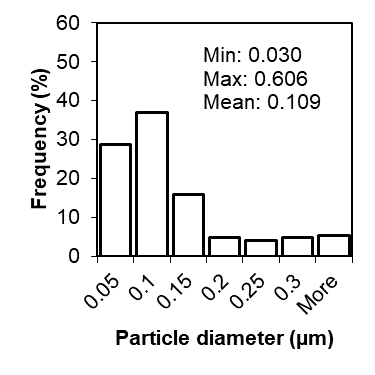 |
| --- | --- |
|  |  |
| 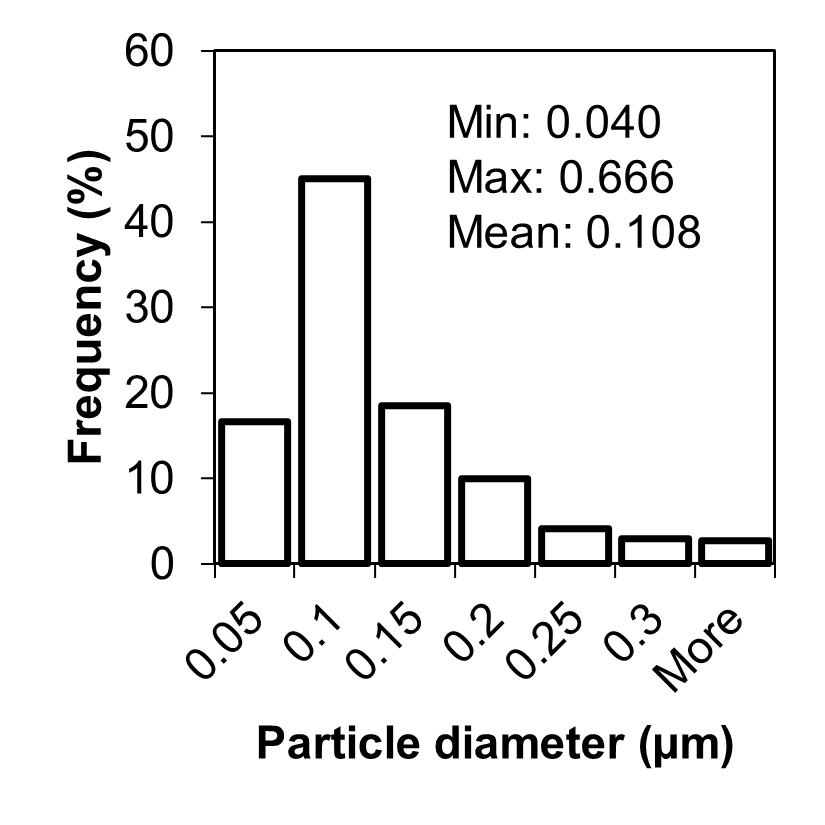 | 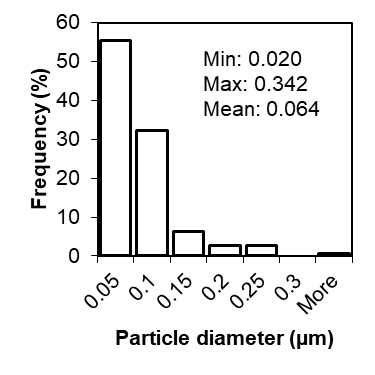 |
|  |  |

**Figure S4.** Nanoparticle size distributions on the modified membranes: a) PVDF-PANI, b) PVDF-PDA, c) PVDF-PANI-TiO_2_, and d) PVDF-PDA-TiO_2_ (TiO_2_ coating concentration of 1 ppm for 2h)

| 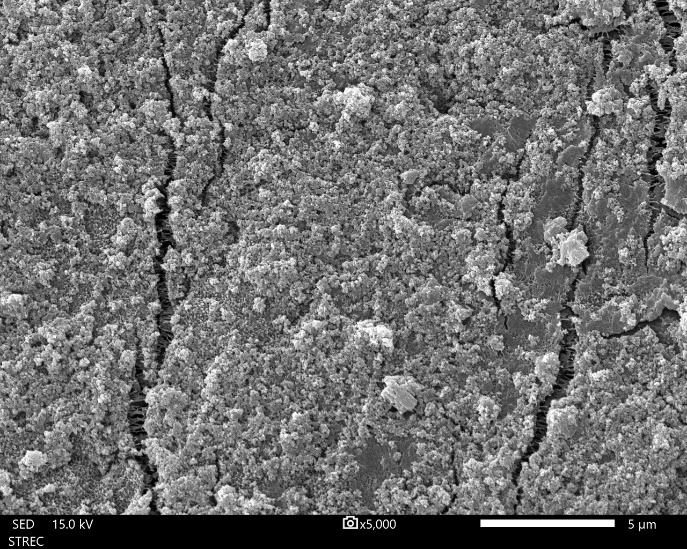  1.24 µm  2.02 µm  1.49 µm | 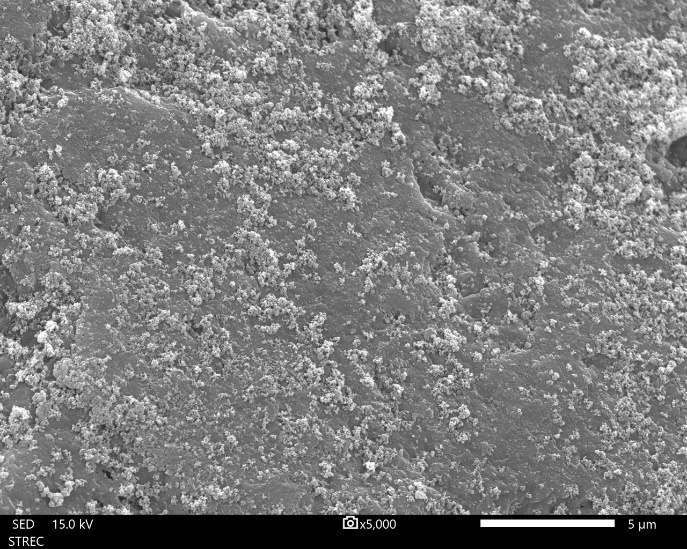 |
| --- | --- |
|  |  |

**Figure S5.** SEM images of modified membrane surfaces at a magnification of 5000×: a) PVDF-PANI-TiO_2_ and b) PVDF-PDA-TiO_2_

|  | C-K | N-K |
| --- | --- | --- |
|  | 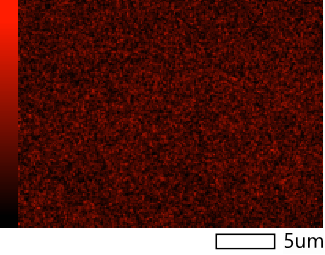 |  |
| O-K | F-K | Ti-K |
|  | 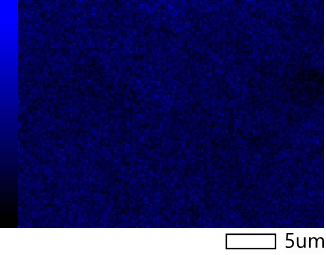 |  |
|  |  |  |


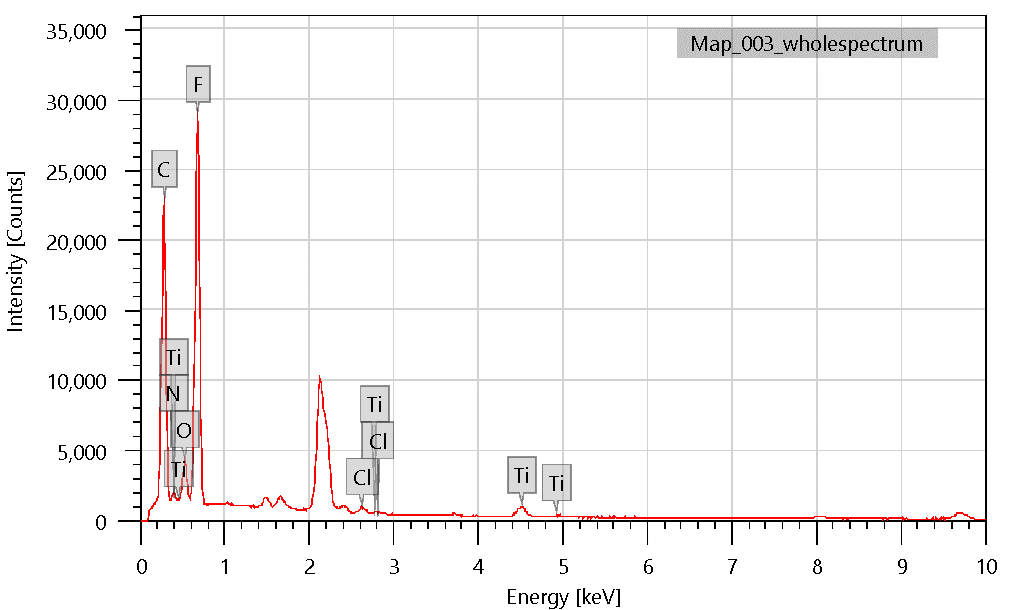


**Figure S6.** Elemental mapping of a PVDF-PANI-TiO_2_ membrane (1 g/l, 2 h)

|  | C-K | N-K |
| --- | --- | --- |
|  |  |  |
| O-K | F-K | Ti-K |
|  | 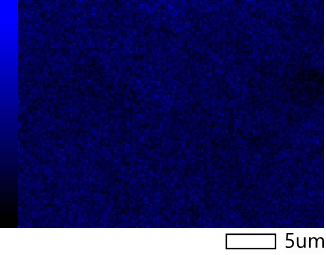 | 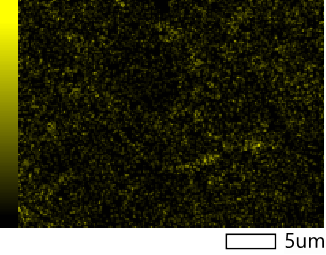 |


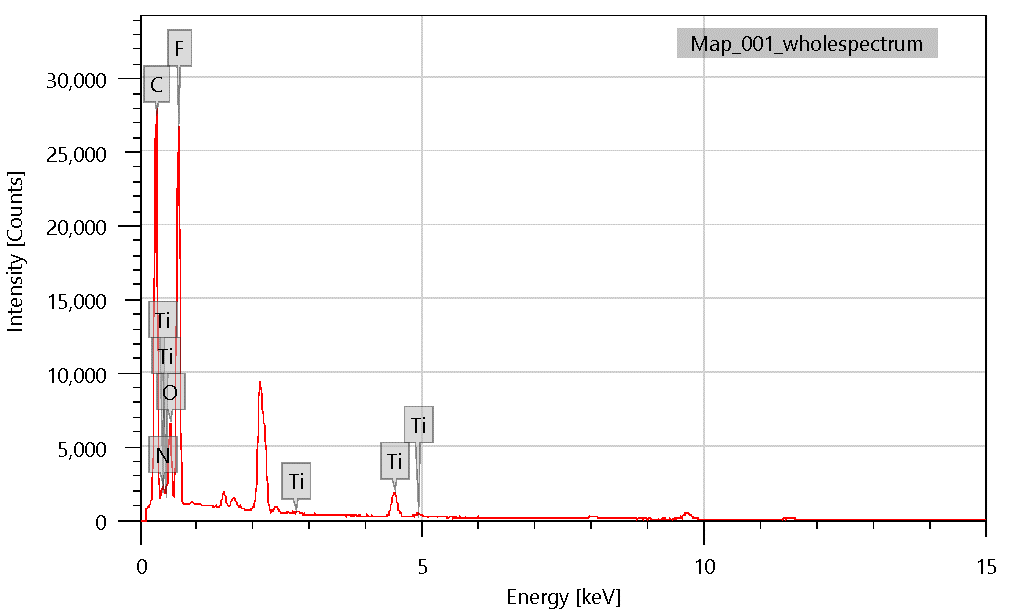


**Figure S7.** Elemental mapping of a PVDF-PDA-TiO_2_ membrane (1 g/l, 2 h)


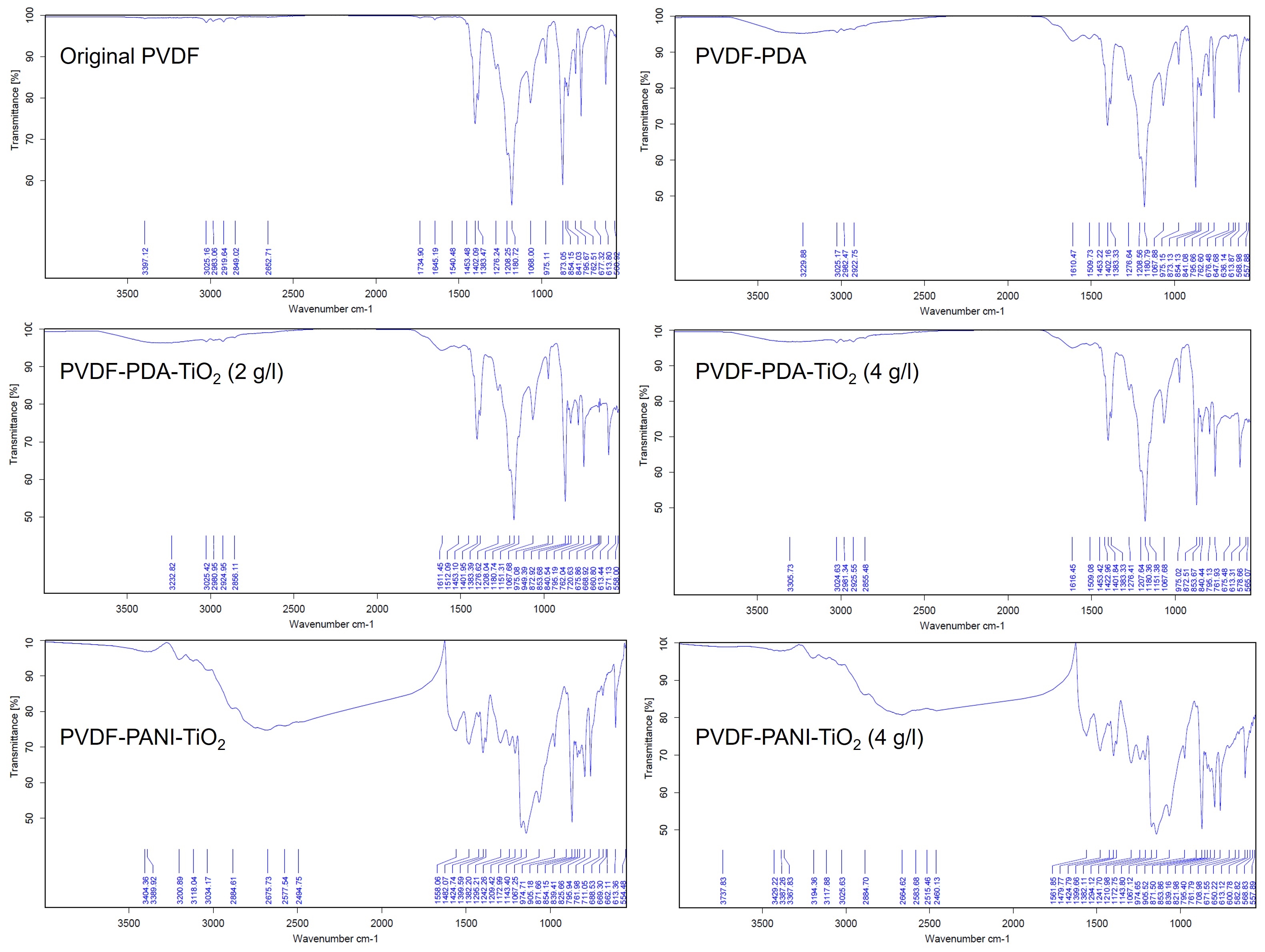


**Figure S8.** FT-IR spectra of original and modified membranes

|  |  |
| --- | --- |
|  |  |
|  |  |

| Parameters | R_a_  (nm) | Diameter of peak (µm) | Distance between peaks (µm) | Peak height (nm) |
| --- | --- | --- | --- | --- |
| Original | 110.8 | 5.05 | 3.8 | 304.2 |
| PVDF-PANI-TiO_2_ | 137.7 | 1.16 | 0.97 | 444 |
| PVDF-PDA-TiO_2_ | 138.9 | 1.15 | 0.88 | 138 |

**Figure S9**. Cross-sectional profiles obtained by atomic force microscopy, performed along the marked directions: a) original membrane b) PVDF-PANI-TiO_2_ membrane, and c) PVDF-PDA-TiO_2_ membrane

| 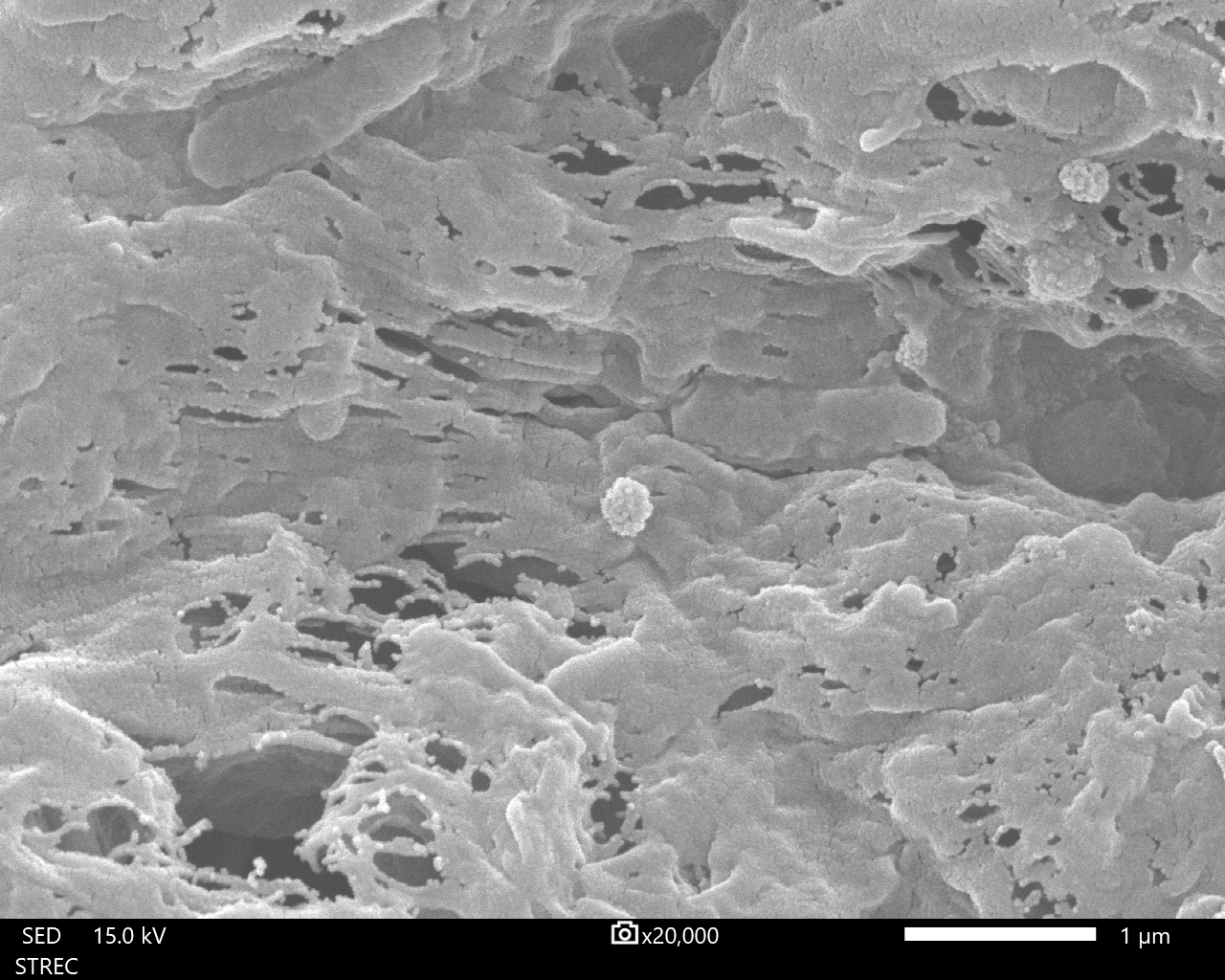 | 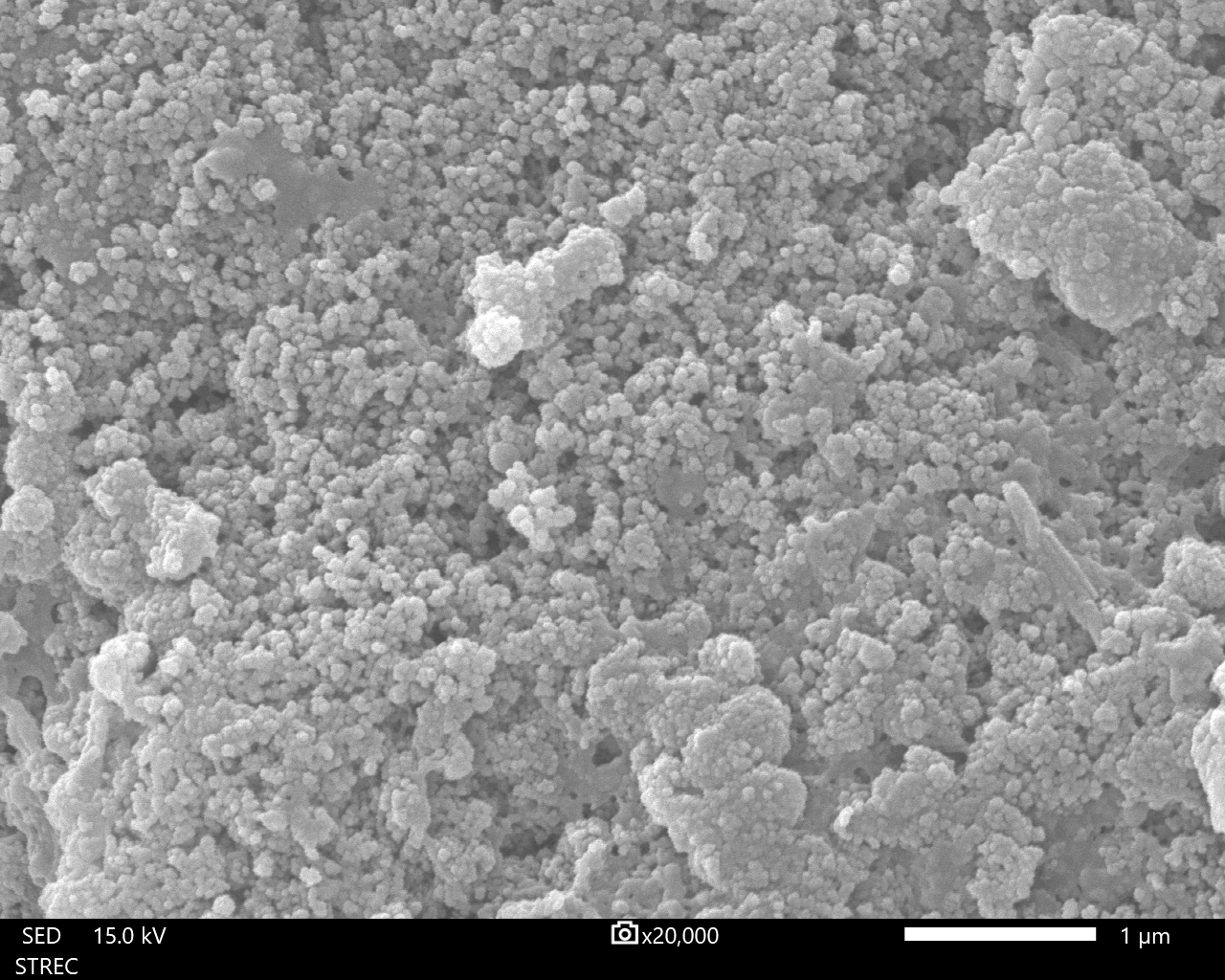 | 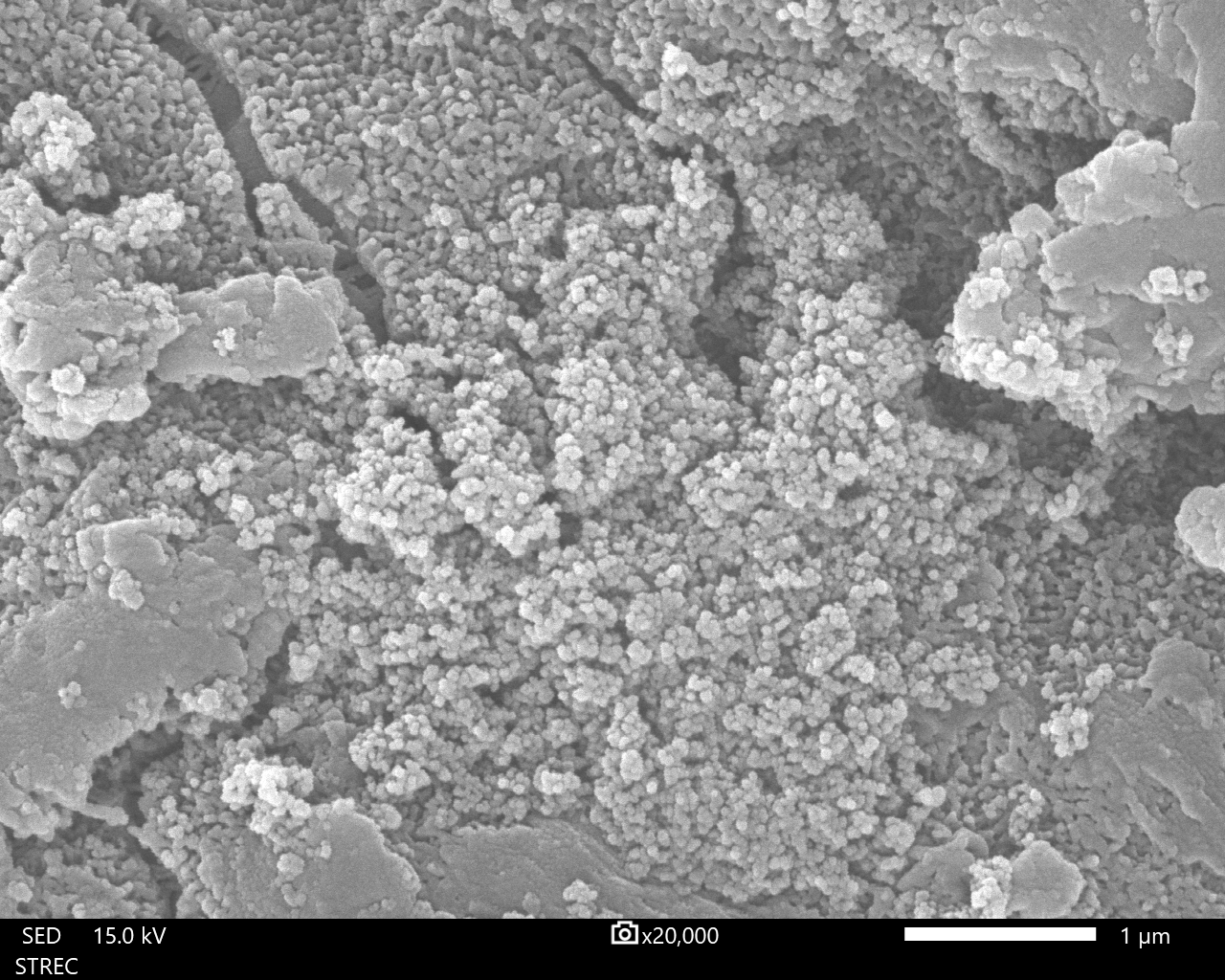 |
| --- | --- | --- |
| PVDF-TiO_2_ | PVDF-PDA-TiO_2_ | PVDF-PANI-TiO_2_ |

**Figure S10.** Changes on membranes after usages (6 h)

1. Le T M H, Singto S et al (2021) Hydrophobic PVDF hollow fiber membrane modified with pulse inductively coupling plasma activation and chloroalkylsilanes for efficient dye wastewater treatment by ozonation membrane contactor. J. Membr. Sci. 119443

2. Le T M H, Wang R, and Sairiam S (2023) Self-protecting PVDF-PDA-TiO_2_ membranes towards highly efficient and prolonged dye wastewater treatment by photocatalytic membranes. J. Membr. Sci. 121789

3. Babar D G, Olejnik R et al (2016) High sensitivity sensor development for Hexamethylphosphoramide by polyaniline coated polyurethane membrane using resistivity assessment technique. Measurement 72-77

4. Ho C-C and Ding S-J (2014) Structure, properties and applications of mussel-inspired polydopamine. Journal of biomedical nanotechnology 3063-3084
